# 3D Structural Fluctuation of IgG1 Antibody Revealed by Individual Particle Electron Tomography

**DOI:** 10.1038/srep09803

**Published:** 2015-05-05

**Authors:** Xing Zhang, Lei Zhang, Huimin Tong, Bo Peng, Matthew J. Rames, Shengli Zhang, Gang Ren

**Affiliations:** 1 The Molecular Foundry, Lawrence Berkeley National Laboratory, Berkeley, CA 94720, USA; 2 Department of Applied Physics, Xi’an Jiaotong University, Xi’an, Shaanxi 710049, China

## Abstract

Commonly used methods for determining protein structure, including X-ray crystallography and single-particle reconstruction, often provide a single and unique three-dimensional (3D) structure. However, in these methods, the protein dynamics and flexibility/fluctuation remain mostly unknown. Here, we utilized advances in electron tomography (ET) to study the antibody flexibility and fluctuation through structural determination of individual antibody particles rather than averaging multiple antibody particles together. Through individual-particle electron tomography (IPET) 3D reconstruction from negatively-stained ET images, we obtained 120 *ab-initio* 3D density maps at an intermediate resolution (~1–3 nm) from 120 individual IgG1 antibody particles. Using these maps as a constraint, we derived 120 conformations of the antibody via structural flexible docking of the crystal structure to these maps by targeted molecular dynamics simulations. Statistical analysis of the various conformations disclosed the antibody 3D conformational flexibility through the distribution of its domain distances and orientations. This blueprint approach, if extended to other flexible proteins, may serve as a useful methodology towards understanding protein dynamics and functions.

Understanding how proteins function in isolation and in their native context requires merging several molecular-level techniques that explore the interplay of protein structure and dynamics^1^. However, current structural determination tools such as X-ray crystallography and single-particle reconstructions often reveal a single unique structure in which protein conformational flexibilities and dynamics are often absent. This is a result of the averaging process, in which thousands to millions of protein molecules assumed to share a single conformation are averaged together in order to enhance signal from proteins and to achieve a common structure. In these methods, the positions of the flexible portions are often averaged out, resulting in a certain degree of information loss on protein conformational flexibility. To disclose the flexibilities or structures of highly dynamic and flexible proteins such as antibodies or lipoproteins, structural determination of each individual protein particle would be required.

Transmission electron microscopy (TEM) serves as a tool for individual protein imaging at atomic resolution, while electron tomography (ET) images an individual protein particle from a series of tilting angles. The first 3D reconstruction of an individual protein particle, fatty acid synthetase, was reconstructed in 1974 by Walter Hoppe and his colleagues through aligning and merging tilted images acquired from a negatively-stained sample^2^,^3^. However, the reconstruction was suspected to be invalid because it was thought that the protein molecule would have been destroyed by the electron beam before it received a sufficient exposure/dose for a validated 3D reconstruction.

Even though a few reconstructions of individual molecules had been reported after Hoppe^3^,^4^,^5^,^6^,^7^,^8^,^9^,^10^,^11^, whether a meaningful resolution structure could be produced from an individual protein particle was still widely suspected. Recently, we reported a method for 3D reconstruction of an individual protein particle, named individual-particle electron tomography (IPET) reconstruction^12^. For a proof-of-concept, we applied this method and reconstructed a few 3D structures at an intermediate resolution (~1–4 nm) from both negative-staining and cryo-electron microscopy samples^10^,^12^.

In this study, we further employed this IPET method to study the dynamics of one of the most well-known flexible proteins: the IgG1 antibody. Through particle-by-particle 3D reconstructions, we reconstructed a total of 120 density maps at an intermediate resolution from negatively-stained ET images. By flexibly docking the crystal structure onto these 3D reconstruction maps, we subsequently achieved 120 conformations of the antibody particles via targeted molecular dynamics (TMD) simulations^13^. The distribution of domain locations and orientation of conformations provided the basis for statistical analysis of antibody flexibility and dynamics.

## Results

### Negative-staining images and reference-free class averages of IgG1 antibody

**Figure 1:**
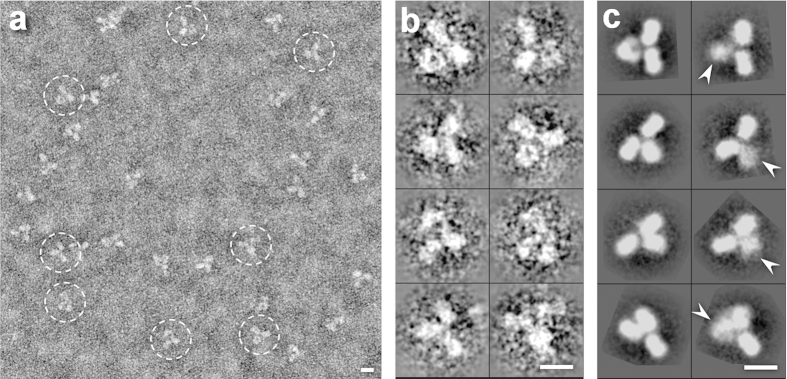
**Negative-staining images and single-particle three-dimensional (3D) reconstructions of IgG1 antibody. a,** A survey of various antibody particles (dashed circles) prepared by optimized negative-staining. The “Y”-shaped particles contained three globular circular or oval domains. **b**, Eight representative individual particles of antibody. **c**, Eight representative reference-free class averages (selected from 80 classes) showed some domains (arrows) were fuzzy or blurry in density. The scale bar was 100 Å.

Imaging of IgG1 antibody (molecular mass ~150 kDa) was performed by optimized negative-staining (OpNS) EM technique^14^,^15^, instead of electron cryo-microscopy (cryo-EM). Cryo-EM often poses a challenge in imaging proteins with molecular masses less than 200 kDa. The survey image (after being Gaussian low-pass filtered to 20Å) showed evenly distributed antibodies having a “Y” shape with dimensions of ~150–180 Å (circles in Fig. 1a, and squares in Supplementary Video). Most antibody particles contained three “ring-shaped” domains of ~55–75 Å in diameter (Fig. 1b and Supplementary Fig. 1a), which corresponded to two F_ab_ domains and one F_c_ domain. The domain sizes and shapes were similar to those of the corresponding crystal structures (PDB entry, 1IGT^16^, 1IGY^17^, 1HZH^18^), suggesting that antibody domains could directly be visualized by OpNS EM technique. The reference-free class averages from 11,373 particles confirmed a “Y”-shape structure (Fig. 1c). However, about half of the class averages were fuzzy or blurry in one or two domains. The blurry domains were due to the protein containing flexible domains (arrows indicated in Fig. 1c and Supplementary Fig. 1b), suggesting the protein was unsuitable for single-particle 3D reconstruction. However, if we ignored these flexibilities and enforced to conduct the conventional approach for 3-dimensional (3D) reconstruction, *i.e.* single-particle reconstruction methods, the 3D reconstructions refined from two sets of initial models showed the final 3D reconstructions contained artifacts in domain structures such as domain size (detailed description in the discussion section and Supplementary Fig. 2).

### 3D reconstruction by individual-particle electron tomography (IPET)

IPET is an EM method for determining an *ab-initio* 3D structure of a single and individual particle of protein from a series of tilt images by electron tomography (ET). Compared to conventional ET 3D reconstruction methods that use large-size whole-micrographs, IPET demonstrated the adverse effects from image distortion and the measuring tilt-errors (including tilt-axis and tilt-angle errors), both of which play a major role in limiting the reconstruction resolution. The method includes a “focused electron tomography reconstruction” (FETR) algorithm to improve the resolution by decreasing the reconstructed image size so it contains only a single-instance protein. As a result, it can tolerate certain levels of image-distortion and tilt-errors, but can precisely determine the translational parameters via an iterative refinement process that contains a series of automatically generated dynamic filters and masks. FETR does not require an assumption that particles share a common structure or a pre-given initial model to study protein structures and dynamics. The use of IPET/FETR could potentially avoid some problems in single-particle reconstruction methods, such as initial model bias, uneven distribution of resolution, or absence of flexible domains.

**Figure 2:**
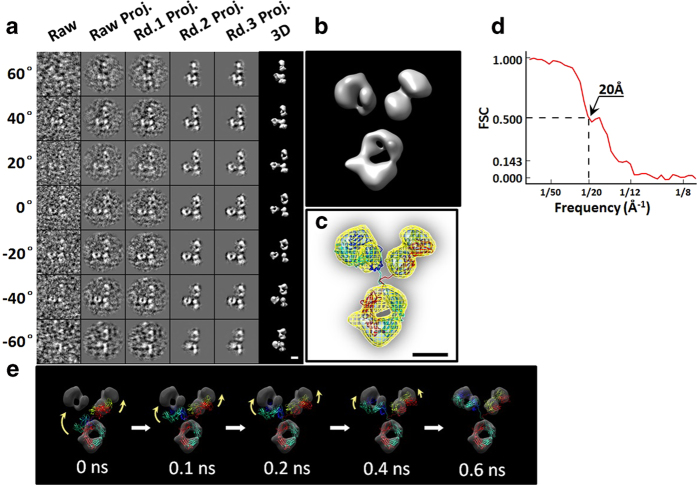
**3D reconstructions of an individual IgG antibody particle by individual-particle electron tomography (IPET)**. **a**, An individual IgG antibody particle was targeted and selected from a tilt series of OpNS-ET micrographs (81 tilt angles, tilt angles from −60° to +60°, in steps of 1.5°). Seven representative tilted views of the targeted particle (selected from 81 tilt micrographs after CTF correction) are displayed in the first column from the left. During the process of IPET reconstruction, the tilting images were gradually aligned to a common center (two translational parameters, x and y) for 3D reconstruction via an iterative refinement process. In round 1 (rd. 1) iterations, a set of automatically generated circular soft-boundary masks was sequentially used. In round 2 (rd. 2), an automatically generated particle-shaped soft-masks were sequentially applied. The round 3 (rd. 3) repeated the rd. 2 iteration with its final mask under an interpolation condition. The projections of the intermediate and final 3D reconstructions at the corresponding tilting angles are displayed beside the raw images in the next six columns. **b**, The final 3D density map of the targeted individual antibody particle reconstructed by IPET. **c**, The final map (displayed in two iso-surfaces) provided a constraint to dock the crystal structure flexibly to achieve a new conformation of antibody via targeted molecular dynamics (TMD) simulations. **d**, Fourier shell correlation (FSC) curve were computed by calculating the cross-correlation of the two density maps reconstructed independently from the two halves (odd and even numbers) of original tilting images (before alignment). The spatial frequency at which FSC fell below 0.5 (instead of 0.143) was modestly used to represent the resolution of the final 3D reconstruction. e, Five snapshot images during TMD simulation illustrated the process of flexibly docking the crystal structure (PDB: 1IGT) into the IPET density map to achieve a new conformation of antibody. During this process, the domain crystal structure remained unchanged (as a rigid body). However, the two flexible linkers were allowed to change in structure, but still maintained proper chemical geometry and bonds. The scale bar was 50 Å.

Although the signal to noise ratios (SNR) of an individual antibody particle in each tilt image (from −60° to + 60° in a step of 1.5°) were only ~0.1 to ~0.26 in the raw micrographs, the overall shape of each individual antibody particle was still visible (Supplementary Video). The images of a targeted antibody particle were iteratively aligned to a global center for an *ab-inito* 3D reconstruction with IPET (Fig. 2 and Supplementary video). During the iterations, the SNRs in the 3D projections were gradually increased to ~1.3 to ~1.8 in the final 3D reconstruction. The dual iso-surfaces of the final 3D reconstruction (displayed in their contained volumes corresponding to 0.6 and 1.6 times of the antibody mass of ~150 kDa) showed three globular densities (two of which were ~60 Å and one ~70 Å in diameter), forming a “Y” shaped structure with an overall diameter of ~150–180 Å. (Fig. 2b).

### A new conformation of IgG antibody

Although the density map resolution from IPET 3D reconstruction was insufficient towards identification of antibody atomic structure as well as inner domain conformational changes, the map was sufficient towards defining the location and orientation of each domain. By rigid-body docking a crystal structure (such as 1IGT^16^) into the IPET reconstruction, the F_c_ domain density map (criteria for domain identification described in supplemental information) showed a near perfect match to that of its crystal structure in both of size and shape (Fig. 2e left panel). However, the two F_ab_ domain density maps were not consistent with the F_ab_ domain crystal structures in both location and orientation (Fig. 2e left panel), suggesting that this current structure determined from this individual antibody had a completely different conformation from that of the antibodies in crystal form. In light of this conformation, we used the IPET reconstruction map as a constraint for refinement of the crystal structure to achieve a new conformation of the antibody through a targeted molecular dynamics (TMD) simulation technique^13^ (Fig. 2c,e, and Supplementary video).

### Resolution analysis on IPET 3D reconstruction

The resolution of IPET reconstruction was estimated by the following four methods: i) a conventional single-particle method^19^,^20^, in which the final aligned tilt images were split into two groups based on their even and odd numbers of tilt order. The images in each group were back-projected into a 3D density map. The Fourier shell correlation (FSC) computed from these two 3D maps was plotted against the spatial frequencies, in which frequencies at which the FSC curve fell below 0.5 were used to represent the reconstruction resolution^19^,^20^. By this method, the resolution was ~16 Å (Supplementary Fig. 3). ii) A gold standard method^21^. To prevent the overestimation of resolution induced by noise correlation in above method (since the tilt images were all aligned to a common 3D reconstruction before back-projected into two 3Ds)^22^, the raw tilt images, rather than the aligned tilt images, were split into two groups based their even/odd tilt order. Two groups of tilt images were independently submitted for IPET 3D reconstruction. The FSC curve between these two final 3D reconstructions was computed, and the frequency at which the FSC curve fell below 0.143 was used as the resolution^21^, i.e. ~13 Å (Supplementary Fig. 3). iii) A method to compare the flexibly fitted crystal structure. The best fitted structure obtained by above TMD method (Fig. 2c and e) was used to generate a 3D density map by *pdb2mrc* software (EMAN package)^23^. The FSC curve between this model’s generated density map and the final IPET reconstruction was computed, and the frequency at which the FSC curve fell below 0.5 was used as the resolution, i.e. is ~29 Å (Supplementary Fig. 3). Considering the crystal domains were rigid-body docked into each domain density maps, the lower resolution measured by this method seems reasonable. iv) A structural feature comparison method. The 3D reconstruction demonstrated some structural features, such as the domain sizes and the “hole” dimensions. These observed structural features could be used to define the resolution. Figure 2c showed the F_c_ domain of crystal structure was well matched to the F_c_ domain of the final 3D reconstruction in domain shape and size as well as its contained “hole”. The crystal structure showed the “hole” diameter in the F_c_ domain is ~25 × ~30 Å, which was surrounded by heavy chain CH domains (CH_2_ and CH_3_) in diameters of ~18 × ~21 × ~30 Å. A reasonable match in size and shape to the 3D reconstruction suggested our reconstruction confirmed that the resolution was within above measured range, i.e. ~20 Å to ~30 Å. Despite the different strategies in determination of the resolution, a quantitative method to estimate the resolution used here was that, we bought the strategy of gold standard method to estimate our IPET map resolution, however, the frequency at which the FSC curve fell below 0.5 (instead of 0.143), i.e. ~20 Å, was used as the resolution (Fig. 2d).

### 120 new conformations of the IgG antibody

**Figure 3:**
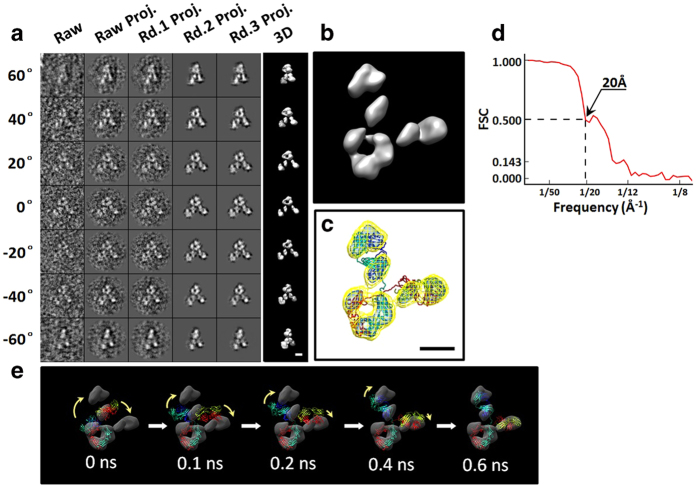
**Detailed procedures of another representative IPET reconstruction.** Detailed IPET reconstruction of another particle was presented here. **a**, Seven representative tilted views of an individual IgG antibody particle shown in the first column (SNR: 0.21), followed by the corresponding projections of 3D reconstruction from major iterations (second to fifth columns), with the final result from each tilting angle shown in the last column. **b**, The finalized 3D reconstruction image (SNR: 1.47) of a targeted individual antibody eventually obtained after above process. **c**, 3D density map and pseudo structure. **d**, FSC curve of IPET reconstruction. **e**, Five snapshot images illustrated the ease of TMD simulation. Molecular Dynamic simulation was used to steer the three domains with a force calculated to match ultimately the three domain density maps in an aqueous environment. The scale bar was 50 Å.

**Figure 4:**
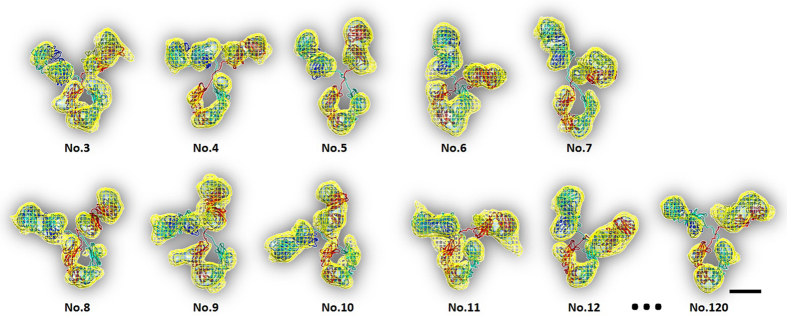
**A zoom-in gallery of eleven representative 3D density maps of antibody reconstructed by IPET.** 11 representative 3D density maps (selected from 120 final 3D density maps of antibody) were displayed in two contour levels. The volume of the inner iso-surface map corresponded to 0.6 times of an IgG1 molecular mass (150kDa), while the volume of outer iso-surface map corresponded to 1.6 times of its mass. By using the EM density maps as constraints, the crystal structure was flexibly docked onto these EM density maps by TMD simulations. During this process, the domain crystal structure remained unchanged; however, two linking loops were changed under condition of energy minima. A total of twelve conformations were achieved. PDB was represented by ribbon and chains were shown by colors.

**Figure 5:**
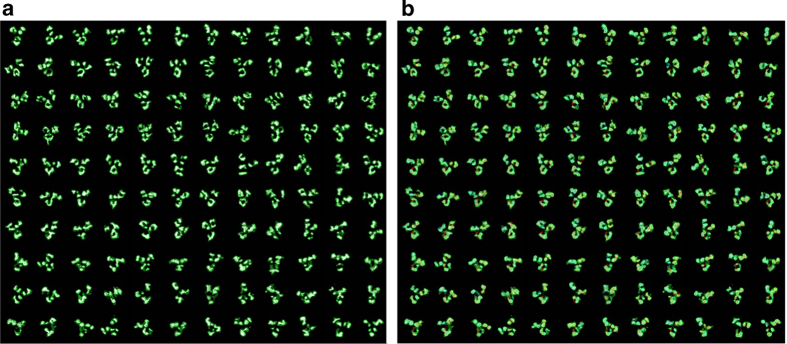
**An overall gallery of a total 120 density maps from IPET reconstruction. a,** The density maps were presented in double contours. Inner contoured (0.6 times IgG1 molecular mass of 150kDa) surface were colored in light gray; outer contours (1.6 times IgG1 molecular mass of 150kDa) were shown by green mesh. **b**, By showing the green mesh with 10% transparency on each map, pseudo PDB structure was represented by ribbon structure and immunoglobulin heavy and light chains were represented by different colors.

Through particle-by-particle 3D reconstructions by IPET, a total of 120 antibody particles were reconstructed (Fig. 3, 4, 5a and Supplementary Fig. 4-14) from a pool of ~300 targeted particles (selected from ~18 sets of tomography data). Certain particles were excluded due to either particle-particle overlapping at certain tilt angles, missing tilted views, unevenly stained surrounding backgrounds, or indistinguishable F_ab_/F_c_ domains. The 120 reconstructions (the resolutions of which were all better than ~20 Å) at two contour levels were displayed after alignment based on their F_c_ domains, and then put into arrays (Fig. 5a). The F_c_ domain of each antibody particle was similar in size and shape to that of the crystal structure, while the F_ab_ domains varied widely in their locations and orientations. By flexibly docking the crystal structure into the EM density maps via TMD simulations, we obtained a total of 120 new conformations (Fig. 2c, 3c, 4, 5b, Supplementary Fig. 4-14e and Supplementary Video). ~50% of the both F_c_ and F_ab_ domains from the EM maps did not completely cover the crystal structure, or were partially different from the crystal structure (Fig. 5b). This might be due to conformational flexibly of domain itself, uneven negative-staining, noise, or reconstruction errors. However, considering that these mismatched portions generally contained less than 25% of the domain volume, these defects did not affect the determination of domain overall location and orientation substantially, and the conformations of each antibody could still be defined from these maps.

### Statistical analysis of the conformational flexibility of IgG1 antibody

**Figure 6:**
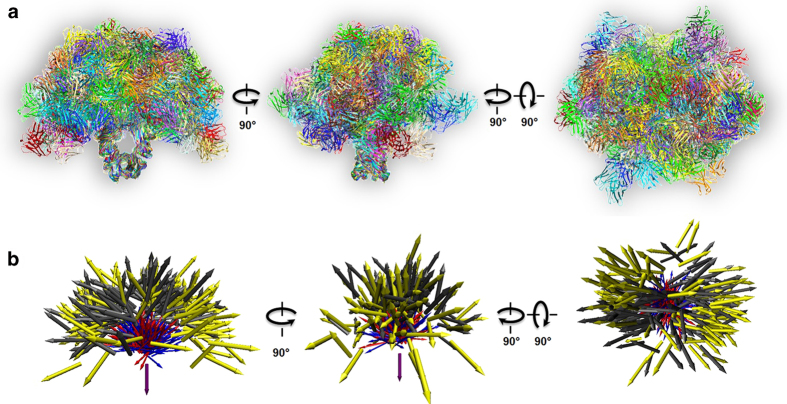
**Conformational flexibility and dynamics of IgG1 antibody. a,** 120 conformations of antibody were aligned together based on their F_c_ domains, and displayed together in three orthogonal views. **b**, Vectors were used to simplify and describe antibody conformations. Information on domain positions and directions from all 120 antibody structures (shown in Fig. 5a) were included. All structures were aligned to domain Fc. Domain Fc was represented as a purple vector. Two Fab domains were represented as yellow and gray vectors. The linker regions (from chain B or D, amino acid residues 221 to 236) were represented as blue and red vectors.

By aligning the 120 conformations along their F_c_ domains, the overlaid reconstructions showed a tree-like structure, in which the F_ab_ domains were distributed over a hemi-ellipsoidal region (Fig. 6a, and Supplementary video), but varied in positions and orientations (Fig. 6b). These variations reflected the conformational flexibility and dynamics of this specific class of antibody.

**Figure 7:**
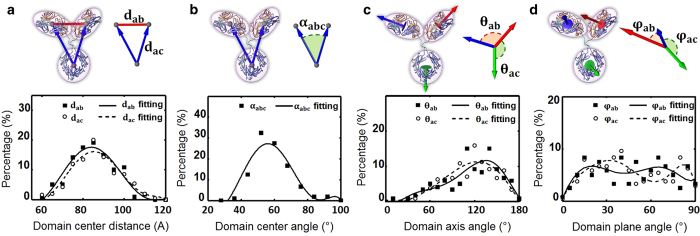
**Distributions of IgG1 antibody domain distances, directions and orientations. a,** By measuring the distance between two corresponding domain centers of each antibody molecule, the histogram of the distances of F_ab_-F_ab_ and F_ab_-F_c_ domains were fitted with sixth degree polynomial curves respectively. **b**, By measuring the angle of F_ab_^1^-F_c_-F_ab_^2^ domains, the histogram was displayed and fitted with sixth degree polynomial curves. **c**, By measuring the angles between the directions of two domains, the histograms of the angles of F_ab_-F_ab_ and F_ab_-F_c_ domains were displayed respectively and fitted with a sixth-degree polynomial curve each. **d**, By measuring the angles between the plane normal directions of each two domains, the histogram of the angles of F_ab_-F_ab_ and F_ab_-F_c_ domains and their corresponding sixth degree polynomial fitting curves are displayed respectively.

Statistical analysis of the antibody conformational flexibility was performed using the four following aspects: i) Histogram of the distances among the domains. The distances between two domains in each molecule were measured based on the mass center of each domain. The results showed that all distances were within a range of 60 to 120 Å. This distance was consistent with that measured by liquid AFM based on the progression of antibody moving on a virus covered flat surface^24^. However, the distance at peak population was ~82.0 ± 2.5 Å (~17.6% of population) for two F_ab_s and ~85.0 ± 2.5 Å (~16.1% of population) for F_ab_ to F_c_ (Fig. 7a), suggesting that the most common antibody conformation had their three domains nearly evenly spaced away from each other in an equilateral triangular shape.

(ii) Histogram of the angles of F_ab_^1^-F_c_-F_ab_^2^ domains showed that over 90% of the antibodies had an angle within a range from ~40° to ~72°, in which the angle at peak population (~28.8%) was ~56.6 ± 4° (Fig. 7b).This finding suggested that the equilateral triangular shape of the antibody had the lowest energy state under the experimental conditions of this presented research. This peak population angle (~60°) was similar to the angle of IgA_1_ revealed by X-ray and neutron scattering^25^, but different from the angle of ~180° of IgD^26^ and IgA_1_^27^ revealed by X-ray and neutron scattering from other groups. The F_ab_^1^-F_c_-F_ab_^2^ angle distribution range was significantly wider than the angle measured from 5 cryo-ET 3D reconstructions of the IgG2a antibody^28^.

(iii) Analysis of the angles between any two domain directions (Fig. 7c). The domain direction was determined by the domain minimum principal axis of inertia as computed by visual molecular dynamics (VMD)^29^. The peak-population angle between two F_ab_ domains was ~135.4° ± 5°, whereas over 70% of the angles were distributed within the full width, starting from the half maximal point of 90^o^ (a range from ~90° to ~160°). This range was consistent with that of AFM measurements from 3D structures of ten antibody particles^30^. Considering the peak-population angle between F_ab_-F_c_ domains was ~124.7° ± 5° (Fig. 7c) and ~135.4° ± 5° for two F_ab_ domains, the ~120° between any two domains suggested that all domains usually stayed in an extended state, probably for maximizing the paratope thermal motion and increasing the chance to bind to epitopes. This result is inconsistent with to that F_ab,_ and F_c_ domains naturally had non-covalent interactions in an IgG antibody^31^. The statistics suggested the optimized distance for one antibody simultaneously binding to two epitopes was ∼130 Å.

(iv) Histogram of the dihedral angles between the normal planes of direction of two domains (Fig. 7d). The normal direction was determined by the domain maxima principal axis of inertia as computed by VMD^29^. The distribution showed that the angles between any two domains were nearly evenly distributed from 10° to 90°. (Since the heavy chain and light chain in F_ab_ domains could not be distingushed within the current resolution, the dihedral angle was measured only up to 90°). A drop in distribution below 10° (Fig. 7d) suggested that the domains normally did not stay parallel to each other (which might be a consequence of long term intermolecular interactions or steric relationships).

## Discussion

### The limitation of single-particle reconstruction on flexible protein

To achieve a 3D structure of a non-crystalized protein, a single-particle reconstruction method was often used. In this process, images from thousands to hundreds of thousands of randomly orientated particles that are assumed to have an identical structure were classified based on projections of a 3D model and then averaged together before being back-projected into a 3D density map via an iterative refinement process^23^. For rigid-body proteins, the single-particle reconstruction is still the best choice for EM to study protein structure without crystallization. However, for structurally flexible proteins, the single-particle reconstruction method has its difficulties and limitations.

To demonstrate the limitations of single-particle reconstruction on a structurally highly flexible protein, we attempted using the single-particle reconstruction to study antibody structure with the EMAN software package^23^ as an example, since the single-particle reconstructions were not significantly different than using different software packages or versions. Two types of initial models were used: featureless blobs and Gaussian blobs^23^. The featureless blob initial models consisted of three spherical blobs which formed a “Y” shape, but with different angles between the two “arms” (Supplementary Fig. 2a). Gaussian blob initial models contained five featureless ellipsoidal Gaussian blobs which each contained different levels of noise (Supplementary Fig. 2b). By a multi-reference refinement process, the final 3D reconstructions refined from the first set of initial models (three “Y” shaped featureless blobs) showed a similar shape and angle (the angle of F_ab_^1^-F_c_-F_ab_^2^) to their initial models (Supplementary Fig. 2a), suggesting the bias from initial model was significant. Moreover, the resulting F_ab_ or F_c_ domains were obviously smaller in dimension than those of the crystal structures (PDB entry, 1IGT^16^, 1IGY^17^, 1HZH^18^), or poor in resolution (failure to show the “ring” shape in domain F_c_) and fuzzy, which was likely due to the flexibility of the domains in position and orientation.

The final five single-particle 3D reconstructions from the second set of initial models (five Gaussian blobs) also showed a “Y” shaped structure (Supplementary Fig. 2b, the second row). These shapes were completely different from the initial models, suggesting that the final 3D reconstructions would have had less bias from the initial models. However, by viewing the maps from a perpendicular direction (Supplementary Fig. 2b, the last and second last rows), the maps showed distinguishable differences from the crystal structure (Supplementary Fig. 2c). These maps either contained substantial noise (first three columns in Supplementary Fig. 2b), or clearly had an artifact in overall or domain structures, such as an “O”-shape for the F_ab_ domains (last two columns in Supplementary Fig. 2b). This artifact may be caused by that the 2D projections were not sensitive enough to be used to distinguish between the differences in 3D conformations and orientations, especially for a highly flexible protein that assumes different conformations. Those phenomena are general issues for single-particle reconstructions on highly dynamic proteins in regardless of that software packages or versions that have been used. It is due to the philosophy of single-particle reconstruction made through averaging thousands of different particles, which may contain structural variability.

Similar artifacts, such as the presence of blurry domains in reference-free class averages^32^, unexpectedly smaller diameter of domains in 3D reconstructions, uneven distribution of resolutions^33^, and absence of protein domains/regions^34^ were also shown in previous single-particle reconstructions. For instance, in the recent single-particle cryo-EM reconstruction of TRPV1 showing an atomic structure on the transmembrane domain, its two ankyrin repeated regions were completely absent in the same reconstruction^35^. Considering the single-particle reconstruction method has its limitations in structural determination of highly dynamic regions of proteins, we here proposed that ET may be a more reliable solution to study flexible protein structures.

Another ET approach to reveal protein structure without requiring crystallization was called single-particle electron tomography (SPET)^36^, in which, hundreds to thousands of 3D subvolumes selected from a large-volume and low-resolution 3D reconstruction are aligned and averaged together in order to improve 3D reconstruction resolution. This approach may reduce the error caused by the conformation and orientation determination (since the 3D was from a single object). The averaging among different subvolumes can only reduce the noise portion in the reconstruction, but cannot reduce the errors presented in the original subvolumes, especially for highly flexible proteins; the alignment of those low-resolution and high-noise subvolumes can be effected by the noise-level and reconstruction errors in the subvolumes and classification of the subvolumes can be limited by the continuous changing of the conformation. To demonstrate the potential limitation, we forcibly averaged the aligned 3D density maps of our 120 antibodies. This averaged map showed the similar phenomena as described above for single-particle reconstruction, i.e. F_ab_ domains were obviously smaller in dimension or fuzzy in density (Supplementary Fig. 15). Based on the above reasons, we used the individual particle electron tomography (IPET) for flexible protein structure determination.

### Possibility for structural determination of an individual antibody 3D structure at an intermediate resolution (1–3 nm)

Structural determination of an individual protein molecule was first attempted about four decades ago^2^. So far, only a few 3D reconstructions from a single protein particle have been reported, including fatty acid synthetase^37^, pre-messenger RNP^4^, adenovirus^7^, IgG2a^6^,^28^, CaMKII^9^; as well as an antibody-drug conjugate^10^ and a 17 nm discoidal high-density lipoprotein (HDL)^12^. Whether a biologically meaningful 3D reconstruction of an individual protein can be achieved remains debatable due to numerous limitations/difficulties from current methods, such as, i) radiation limitation^38^; ii) high-noise/low-contrast images; iii) low number of images (~100 images); iv) difficulty in CTF corrections; v) challenging data acquisition at a high magnification (~100 K × ); vi) image distortion; vii) tilt-angle determination errors; and viii) a theoretically calculated limit of resolution at ~20 Å^39^.

We, however, believed in the possibility of achieving a structure of an individual protein structure at near nanometer resolution because: i) nearly all TEM instruments have a resolution capability better than 3 Å; ii) ~100 low-dose images (signal-to-noise ratio of ~0.13) are theoretically sufficient to reconstruct a 3D density map of sub-nanometer resolution^12^; iii) a ~20 Å resolution limit is achievable in principle by using a total dose >5e^−^/Å^2^ 12,39. Practically, to obtain a 3D structure of an individual protein at near nanometer resolution is very challenging. Conventional ET reconstruction methods used large micrographs for 3D reconstruction, since it was believed that larger sized micrographs provided more signal for more accurate determination of the geometric tilt-angles and translation parameters, and therefore helped achieve a 3D reconstruction with a higher resolution. We found the imaging distortions, such as defocus^12^, astigmatism^40^, non-linear projector lens^41^,^42^, pincushion and spiral^43^,^44^, energy filter^45^,^46^, and even radiation-induced deformations^47^,^48^ played a key role in reducing the accuracy of determining the geometric angles. Consequently, the tilt-angle errors affected the accuracy of determining the translational parameters, which directly caused the resolution of 3D reconstructions to decline significantly^49^. Thus, we proposed the IPET methodology, in which the reconstruction size was simply reduced by including only a single-instance molecule for 3D reconstruction^50^. The small image-size limited the translational errors induced by geometric angle errors, therefore limiting the adverse effects of distortions to the 3D reconstruction. Since IPET strategy uses small image-size reconstruction (similar strategy had previously been reported by Cantele etc^51^), IPET reconstruction could tolerate a certain level of tilt angle/axis errors, and achieve an *ab-inito* 3D reconstruction without requiring a pre-given initial model.

In this research, we obtained 120 structures from each individual antibody particle at an intermediate resolution. Despite that the fact that the TEM instrument used in this work was one of lowest models of TEM (LaB_6_ filament) and the micrographs were acquired by a traditional CCD (4,096 × 4,096 pixel Gatan Ultrascan CCD camera, instead of a state-of-art Direct Detection camera), our achieved resolution was benefited by i) IPET/FETR reconstruction strategy/algorithms; ii) sample preparation by high-resolution OpNS protocol^14^,^15^; iii) near-perfect TEM alignment condition (CTF Thon ring reach beyond 5 Å under 1.6 μm defocus^52^); iv) low defocus imaging (< ~1.0 μm); and v) higher dose imaging conditions (~60–200 e^−^/Å^2^).

We, however, encountered the following weaknesses in this current study: i) certain preferred orientation of particles; ii) certain level of protein flatness due to the drying process; iii) uneven staining distribution; iv) unidentified artifacts through negative-staining; v) missing wedge effects (tilted only up to ± 60° instead of ± 90°); vi) inability to identify the heavy and light chains within F_ab_ domains; vii) certain level of inaccuracy in identifying F_ab_ and F_c_ domains; viii) time consuming data acquisition (~2–3 hours spent on each tomographic set of ~100 images); ix) challenges in tomographic data collection under high magnification (~80 K × ); and x) low efficiency in 3D reconstructions. For example, in this study, a total of 30 tilting series were acquired, in which more than half of the tilting series was excluded due to an uneven stain or jumps in defocus (mechanical vibration). Only particles visualized with a clear boundary were selected. As a result, each tilting series contributed 5~40 antibody particles for IPET 3D reconstruction. In total, ~60% of IPET 3D density maps were excluded for structural analyses due to uneven staining, buffer solvent aggregation, tilting images that were missing at certain angles, protein overlapping, deformation, missing alignment, and failure to identify F_ab_ and F_c_ domains in a molecule. Eventually, only ~40% of the IPET maps (a total number of ~120) were submitted for conformational determination by TMD and statistical analyses. In the case of domain identification, IPET 3D reconstructions showed that the F_c_ domain had an overall size similar to that of the crystal structure, but presented a larger center hole and stronger densities than that from the crystal structure. One of the best tilting series showed ~60 particles presented in the 0° tilting micrograph (Supplementary Video). Of these 60 particles, about a third, i.e. 21 particles, contributed to IPET reconstructions and structural analyses. As for the remaining 39 particles, 18 were excluded for structural analyses due to either tilting images missing at certain angles, overlapping views or unclear contour. The other 21 particles were excluded due to difficulties in determining protein conformation, specifically failure to identify the F_c_ and F_ab_ domains or uncertain domain orientation, which may have been caused by uneven staining, protein deformation, negative staining or miss-alignment during IPET reconstruction.

In spite of the above weaknesses, the success in obtaining over a hundred 3D structures at an intermediate resolution from each individual antibody particle (one of most highly dynamics and flexible proteins) demonstrates that IPET could be a useful approach towards achieving a meaningful resolution structure for studying the highly dynamic, heterogeneous and flexible protein particles without involving any averaging of different particles^12^.

## Materials and methods

### EM specimen preparation by the optimized NS (OpNS) protocol

Monoclonal antibody IgG subclass 1 (h38C2, humanized version of m38C2, molecular mass ~150 kDa), was expressed in HEK-293 cells, and purified using protein A/G chromatography^53^. Antibody sample (~20.0 mg/ml) was diluted to ~0.05 mg/ml with Dulbecco’s phosphate-buffered saline buffer (DPBS: 2.7 mM KCl, 1.46 mM KH_2_PO_4_, 136.9 mM NaCl, and 8.1 mM Na_2_HPO_4_; Invitrogen), and then prepared using the optimized NS protocol by 1% (w/v) uranyl formate (UF)^14^,^15^.

### EM micrograph acquisition and single particle analysis

2D data image for single particle analysis of all antibody specimens were acquired under a near Scherzer focus defocus condition (~0.01 μm to 0.15 μm) with a high-sensitivity 4,096 × 4,096 pixel Gatan Ultrascan CCD camera, and at 80,000 x magnification, using a Zeiss Libra 120 TEM. Raw images were low-pass filtered to 10 Å to reduce noise, and high-pass filtered to 600 Å to remove large area background fluctuation. Particles were selected by EMAN Boxer^23^. A total number of 11,373 particles were selected with a box size of 192 × 192 pixels. All particles were masked by a round mask (diameter 180 Å) with Gaussian boundary generated by SPIDER^54^. Particles were divided into 80 classes and averaged images were shown in Fig. 1c and Supplementary Fig.1a. Two groups of initial models were generated for performing single particle reconstruction via multi-reference refinement, respectively. The first group contained three models. Each of these models contained three spheres (their relative sizes corresponded to the three antibody domains, two F_ab_ for 40Å, F_c_ for 50 Å) with a different separation angle (angle F_ab_^1^-F_c_-F_ab_^2^, 30°, 60°, 90°, Supplementary Fig. 2a). The second group contained five models and each of them contained one Gaussian ellipsoid (diameter ~150 × 120 × 90 Å) with different flat band noises added (0, 1.0, 2.0, 3.0 and 4.0 times of image standard deviation using *proc3d* on EMAN software; Supplementary Fig. 2b). Reference free class averages and single particle reconstructions were performed by EMAN^23^.

### Electron tomography data acquisition and image pre-processing

Electron tomography data of all antibody specimens were acquired under similar conditions by same TEM and CCD as described above. The specimens mounted on a Gatan room temperature high-tilt holder were tilted at angles ranging from −60° to 60° in steps of 1.5°. The total dose of electron illumination was ~200 e^−^/Å^2^ The tilt series of tomographic data under defocus ~1.0 μm were controlled and imaged by manual operation using Gatan tomography software (for Zeiss Libra 120 TEM) that had been preinstalled in the microscopes. Micrographs were initially aligned together with the IMOD software package^55^ or UCSF Tomography^56^. The defocus near the tilt-axis area of each tilt micrograph was examined by fitting contrast transfer function (CTF) parameters with its power spectrum using ctffind3 in the FREALIGN software package^57^, followed by *ctfit* (EMAN software package)^23^. The CTF was then corrected by TOMOCTF^58^.

### Selection and identification of antibody particles

Each micrograph of antibody particles was Gaussian low-pass filtered and examined before selection for 3D reconstruction. A small image containing only one targeted particle was selected, and marked with a window size of 220 × 220 pixels (~326 × 326 Å) on the 0° tilt image micrograph^50^. The tilt series of images of targeted particles were automatically tracked and picked out, and then manually checked to remove particles which were missing any tilt image, were overlapped or damaged or were surrounding by uniform stains. Particles with uniform stain condition were identified and selected as targets for IPET 3D reconstruction.

### 3D reconstruction by individual-particle electron tomography (IPET)

In IPET reconstruction, an initial model was obtained via direct back-projection of these small-images into a 3D map, followed by 3D reconstruction refinements performed via three rounds of refinement loops with an FETR algorithm^12^. All IPET density maps presented in figures were low-pass filtered to 20 Å. SNR for each 3D map was calculated using equation SNR = (*I*_S_−*I*_*b*_)/*N*_*b*_, in which *I*_*s*_ was the average power inside particle, and *I*_*b*_ was the average power outside the particle. The standard deviation of noise, *N*_*b*_, was calculated from background standard deviation outside the particle. The particle area was defined by using particle shaped mask generated from IPET final 3D with low pass filtered to 40 Å and set as 3 times its molecular weight. This same method was used to calculate 2D SNR. 2D mask was generated based on the 3D projection at each tilt angle.

### Resolution analysis by Fourier shell correlation (FSC)

To analyze tomographic 3D reconstructions, the raw ET images were split into two groups based on having an odd- or even-numbered index in the order of tilt angles^12^. Each group was submitted for IPET reconstruction respectively to generate a 3D reconstruction; these two 3D reconstructions were subsequently used to compute the FSC curve over their corresponding spatial frequency shells in Fourier space (using the “RF 3” command in SPIDER)^54^. The frequency at which the FSC curve decreased to a value of 0.5 was used to represent the resolution of the final IPET 3D density map^12^. Other two FSC curves, i.e. FSC curve between flexibly fitted crystal structure and IPET final 3D, and FSC curve between two half of tilt images which aligned by IPET, were also used calculated for estimate the resolution.

### Obtaining a pseudo structure by fitting the domains from crystal structure into an IPET density map

The current resolution (~1–3 nm) of IPET 3D reconstructions were not sufficient to determine the secondary structure of each individual antibody; however, the reconstructions were sufficient to provide the information about the domain orientations and positions, which may reveal the domain fluctuation. We chose 1IGT as an example to reflect the antibody dynamics by docking its three domains into each domain in the reconstruction. We chose 1IGT is because there is no full length human IgG1 crystal structure available so far. Three available crystal structures of antibodies, 1IGT, 1HZH and 1IGY are very similar in the lengths of sequences. This small difference would not make a significant difference at a ∼1–3 nm resolution of 3D reconstructions in the analysis of the domain locations and orientations. The F_ab_ and F_c_ domains of the mouse IgG2 antibody were therefore truncated from the crystal structure 1IGT^16^, and then the domains were rigid-body docked into a density map envelope by Chimera^59^. The positions and orientations of the docked domains were saved for performing targeted molecular dynamic (TMD) simulations^13^.

To identify the F_ab_ and F_c_ domains in the 3D reconstruction, the following criteria was used: i) the hole size of F_c_ domain was distinguishably larger than that of F_ab_ domain, and ii) under high contour levels, F_c_ domain density was more like a “C” or “O” shape, while F_ab_ resembled more of a “dumb-bell” shape (Fig. 2b top).

By using the best-fit positions as the target positions, we drove the conformation change to correlate with density map by target molecular dynamic (TMD) simulation technique^13^. The dragging and moving force was pre-calculated and then applied to all the backbone atoms of each domain of crystal structure to gradually steer toward their corresponding best-fit positions and orientations. During this process, the domain structure conformation as well as the chemical structure, including disulfide bonds, was constrained as the original crystal structure, whereas the loop regions were left flexible, allowing the conformational change to occur (Fig. 2e and Supplementary video). As a result, the achieved new structure/conformation of antibody had the same domain structure as in the crystal structure, but differed in the relative position and orientation.

The TMD simulation was performed using the program NAMD2.7b^60^. CHARMM22 force fields^61^ were used for protein, TIP3P water and ions. Periodic boundary conditions were performed for each system and a cut-off distance for van der Waals interactions was set to 12 Å. The Particle-Mesh Ewald (PME) method was applied to evaluate the electrostatic forces of the systems. The whole system was heated from 0 K to 310 K over 62 ps simulation, using weakly coupled Langevin dynamics, and the temperature maintained at 310 K. The pressure was maintained at 1 atm using a Langevin piston Nose-Hoover barostat (with a piston period of 100 fs and a decay time of 50 fs). It took 300k steps to finish the conformation transfer, and simulation length was 0.6 ns. Root mean-square deviations (RMSD) of the protein structure and cross-correlation change (CC C) between the protein and density map were calculated in order to analyze the equilibration state of each trajectory.

### Statistical analysis of antibody dynamics

To determine the structural dynamics of the antibody, all pseudo structures were aligned based on their F_c_ domains using the VMD command^29^. The differences in distance and orientation between the F_ab_ domains were measured and analyzed to study the equilibrium fluctuation and structural dynamic character of antibody particles. In brief, we introduced nine vectors to represent the domain location and orientation with respect to each other (Fig. 7). The domain location was defined as the mass center of each domain, which is as follows: F_ab_^1^, chains A and B from amino acid residues 1 to 226; F_ab_^2^, chains C and D from residues 1 to 226; F_c_, chain B from residues 237 to 444 and chain D from residues 237 to 444. The first three vectors were the domain-domain vectors, d_ab_ and d_ac,_ that were measured from the mass center of one domain to the mass center of other domain; the second three vectors were domain direction vectors that followed the longest direction of each domain; the last three vectors were the normal direction vectors that were perpendicular to the largest projection surface of each domain. We measured the following parameters for statistical analyses: i) the length of each domain-domain vectors; ii) the angle, α_abc_, between two F_c_-F_ab_ domain-domain vectors; iii) the angle between two F_ab_ domain direction vectors; iv) the angle between two domain direction vectors of F_ab_ and F_c_ domains; v) the angle between two normal direction vectors of two F_ab_ domains plane; and vi) the angle between two normal directions of F_ab_ and F_c_ domains plane. These measurements were used to calculate the angles between the domains. The distances and angles between antibody domains were plotted onto histograms and fitted with sixth degree polynomial curves by MATLAB. These histograms represented the conformational space of each antibody molecule, which in turn indicated the extent of its dynamic flexibility.

## Author Contributions

This project was designed and initiated by G.R. and L.Z., and the E.M. specimens were prepared by P.B. and H.T. X.Z., L.Z. and H.T. acquired the E.M. images. X.Z. and L.Z. conducted the image process and 3D reconstruction by IPET, G.R conducted the single-particle reconstruction. X.Z. performed the conformation determination by TMD., and statistically analyzed the distributions. X.Z., S.Z. and G.R. conducted the resolution analysis. X.Z., L.Z., M.J.R. and G.R. interpreted and manipulated the data. G.R. and X.Z. drafted the initial manuscript, which was revised by M.J.R., Z.L., B.P. and H.T.

## Additional Information

**Accession Codes**: Density maps of all 120 antibodies have been deposited at the Electron Microscopy Data Bank (EMDB) and assigned reference codes EMDB-6099.

**How to cite this article**: Zhang, X. *et al.* 3D Structural Fluctuation of IgG1 Antibody Revealed by Individual Particle Electron Tomography. *Sci. Rep.* **5**, 09803; doi: 10.1038/srep09803 (2015).

## Supplementary Material

Supplementary Information

Supplementary Video 1
