# Corrigendum: 3D Structural Fluctuation of IgG1 Antibody Revealed by Individual Particle Electron Tomography

**DOI:** 10.1038/srep17919

**Published:** 2016-01-06

**Authors:** Xing Zhang, Lei Zhang, Huimin Tong, Bo Peng, Matthew J. Rames, Shengli Zhang, Gang Ren

Scientific Reports 5: Article number: 9803; 10.1038/srep09803published online: 05052015; updated: 01062016

This Article contains a typographical error in the Results section under subheading ‘Statistical analysis of the conformational flexibility of IgG1 antibody’.

“The F_ab_^1^-F_c_-F_ab_^2^ angle distribution range was significantly wider than the angle measured from 5 cryo-ET 3D reconstructions of the IgG2a antibody^28^.”

should read:

“The F_ab_^1^-F_c_-F_ab_^2^ angle distribution range was significantly wider than the angle measured from 5 cryo-ET 3D reconstructions of the IgG2a antibody^6^.”

