# Supplementary material for: 3D Structural Fluctuation of IgG1 Antibody Revealed by Individual Particle Electron Tomography
Source: Sci Rep. 2015 May 5;5:9803. doi: 10.1038/srep09803 (PMC4419541; doi:10.1038/srep09803)
Supplement: Supplementary Information [file srep09803-s1.pdf]

Supplementary Information

3D Structural Fluctuation of IgG1 Antibody Revealed by Individual Particle Electron Tomography

Xing Zhang[1,2,†], Lei Zhang[1,†], Huimin Tong[1], Bo Peng[1], Matthew J. Rames[1], Shengli Zhang[2], Gang Ren [1]*

[1]The Molecular Foundry, Lawrence Berkeley National Laboratory, Berkeley, CA 94720, USA
[2]Department of Applied Physics, Xi'an Jiaotong University, Xi'an, Shaanxi 710049, China

[†] These two authors contributed equally to this work

***** To whom correspondence should be addressed:
Phone: (510) 495-2375; E-mail: gren@lbl.gov

Supplementary Figures

Supplementary Figure 1 | 2D raw particles and reference-free class averages of antibody images. a, Raw antibody particles after being Gaussian low pass filtered to 20 Å for a better view. Round shaped mask (diameter, 180 pixels) with Gaussian boundary (~20 pixels) applied. **b**, 80 reference-free class averages from a total of 11,373 particles. The number at the bottom left hand corner of each grid referred to the total number of particles that contributed to the average. The fuzzy domains were indicated by arrows. Bars: 100 Å.

Supplementary Figure 2 | Single particle reconstructions of IgG antibody. Two groups of initial models were used to perform single particle reconstruction. **a**, Multi-model refinement results from initial models of antibody with three globular domains positioned at different relative F_{ab}-F_c-F_{ab} angles. **b**, Multi-model refinement results from initial models of antibody represented by Gaussian ellipsoids with different noise ratio. **c**, crystal structure of mouse IgG2a (1IGT [1]) in different views.

Supplementary Figure 3 | Resolution analysis on IPET 3D reconstruction The resolution of IPET reconstruction was estimated by following three Fourier shell correlation (FSC) curves. The first FSC curved (black solid line) was computed from two 3D maps that were back-projected from two sets of aligned tilt images (with even and odd tilt order numbers). The second FSC curve (gray dashed line) was calculated from two 3D maps that were independently refined / reconstructed from two sets of original/unaligned tilt images (with even and odd tilt order numbers). The third FSC curve (gray line) was computed from the IPET reconstruction and the atomic density map which was calculated from the best fit PDB model.

Supplementary Figure 4 | Detailed procedures of the third representative IPET reconstruction. Detailed IPET reconstruction of particle No. 3 was presented here. **a,** Seven representative tilted views of an individual IgG antibody particle shown in the first column (SNR: 0.28), followed by the corresponding projections of 3D reconstruction from major iterations (second to fifth columns), with the final result from each tilting angle shown in the last column. **b,** The finalized 3D reconstruction image (SNR: 1.49) of a targeted individual antibody eventually obtained after above process. **c,** 3D density map and pseudo structure. **d,** FSC curve of IPET reconstruction. **e,** Five snapshot images illustrated the ease of TMD simulation. Molecular Dynamic simulation was used to steer the three domains with a force calculated to match ultimately the three domain density maps in an aqueous environment. The scale bar was 50 Å.

Supplementary Figure 5 | Detailed procedures of the fourth representative IPET reconstruction. Detailed IPET reconstruction of particle No. 4 was presented here. **a,** Seven representative tilted views of an individual IgG antibody particle shown in the first column (SNR: 0.16), followed by the corresponding projections of 3D reconstruction from major iterations (second to fifth columns), with the final result from each tilting angle shown in the last column. **b,** The finalized 3D reconstruction image (SNR: 1.31) of a targeted individual antibody eventually obtained after the above process. **c,** 3D density map and pseudo structure. **d,** FSC curve of IPET reconstruction. **e,** Five snapshot images illustrated the ease of TMD simulation. Molecular Dynamic simulation was used to steer the three domains with a force calculated to match ultimately the three domain density maps in an aqueous environment. The scale bar was 50 Å.

Supplementary Figure 6 | Detailed procedures of the fifth representative IPET reconstruction. Detailed IPET reconstruction of particle No. 5 was presented here. **a,** Seven representative tilted views of an individual IgG antibody particle shown in the first column (SNR: 0.15), followed by the corresponding projections of 3D reconstruction from major iterations (second to fifth columns), with the final result from each tilting angle shown in the last column. **b,** The finalized 3D reconstruction image (SNR: 1.55) of a targeted individual antibody eventually obtained after the above process. **c,** 3D density map and pseudo structure. **d,** FSC curve of IPET reconstruction. **e,** Five snapshot images illustrated the ease of TMD simulation. Molecular Dynamic simulation was used to steer the three domains with a force calculated to match ultimately the three domain density maps in an aqueous environment. The scale bar was 50 Å.

Supplementary Figure 7 | Detailed procedures of the sixth representative IPET reconstruction. Detailed IPET reconstruction of particle No. 6 was presented here. **a,** Seven representative tilted views of an individual IgG antibody particle shown in the first column (SNR: 0.23), followed by the corresponding projections of 3D reconstruction from major iterations (second to fifth columns), with the final result from each tilting angle shown in the last column. **b,** The finalized 3D reconstruction image (SNR: 1.73) of a targeted individual antibody eventually obtained after the above process. **c,** 3D density map and pseudo structure. **d,** FSC curve of IPET reconstruction. **e,** Five snapshot images illustrated the ease of TMD simulation. Molecular Dynamic simulation was used to steer the three domains with a force calculated to match ultimately the three domain density maps in an aqueous environment. The scale bar was 50 Å.

Supplementary Figure 8 | Detailed procedures of the seventh representative IPET reconstruction. Detailed IPET reconstruction of particle No. 7 was presented here. **a,** Seven representative tilted views of an individual IgG antibody particle shown in the first column (SNR: 0.11), followed by the corresponding projections of 3D reconstruction from major iterations (second to fifth columns), with the final result from each tilting angle shown in the last column. **b,** The finalized 3D reconstruction image (SNR: 1.23) of a targeted individual antibody eventually obtained after the above process. **c,** 3D density map and pseudo structure. **d,** FSC curve of IPET reconstruction. **e,** Five snapshot images illustrated the ease of TMD simulation. Molecular Dynamic simulation was used to steer the three domains with a force calculated to match ultimately the three domain density maps in an aqueous environment. The scale bar was 50 Å.

Supplementary Figure 9 | Detailed procedures of the eighth representative IPET reconstruction. Detailed IPET reconstruction of particle No. 8 was presented here. **a,** Seven representative tilted views of an individual IgG antibody particle shown in the first column (SNR: 0.13), followed by the corresponding projections of 3D reconstruction from major iterations (second to fifth columns), with the final result from each tilting angle shown in the last column. **b,** The finalized 3D reconstruction image (SNR: 1.35) of a targeted individual antibody eventually obtained after the above process. **c,** 3D density map and pseudo structure. **d,** FSC curve of IPET reconstruction. **e,** Five snapshot images illustrated the ease of TMD simulation. Molecular Dynamic simulation was used to steer the three domains with a force calculated to match ultimately the three domain density maps in an aqueous environment. The scale bar was 50 Å.

Supplementary Figure 10 | Detailed procedures of the ninth representative IPET reconstruction. Detailed IPET reconstruction of particle No. 9 was presented here. **a,** Seven representative tilted views of an individual IgG antibody particle shown in the first column (SNR: 0.17), followed by the corresponding projections of 3D reconstruction from major iterations (second to fifth columns), with the final result from each tilting angle shown in the last column. **b,** The finalized 3D reconstruction image (SNR: 1.42) of a targeted individual antibody eventually obtained after the above process. **c,** 3D density map and pseudo structure. **d,** FSC curve of IPET reconstruction. **e,** Five snapshot images illustrated the ease of TMD simulation. Molecular Dynamic simulation was used to steer the three domains with a force calculated to match ultimately the three domain density maps in an aqueous environment. The scale bar was 50 Å.

Supplementary Figure 11 | Detailed procedures of the tenth representative IPET reconstruction. Detailed IPET reconstruction of particle No. 10 was presented here. **a,** Seven representative tilted views of an individual IgG antibody particle shown in the first column (SNR: 0.26), followed by the corresponding projections of 3D reconstruction from major iterations (second to fifth columns), with the final result from each tilting angle shown in the last column. **b,** The finalized 3D reconstruction image (SNR: 1.56) of a targeted individual antibody eventually obtained after the above process. **c,** 3D density map and pseudo structure. **d,** FSC curve of IPET reconstruction. **e,** Five snapshot images illustrated the ease of TMD simulation. Molecular Dynamic simulation was used to steer the three domains with a force calculated to match ultimately the three domain density maps in an aqueous environment. The scale bar was 50 Å.

Supplementary Figure 12 | Detailed procedures of the eleventh representative IPET reconstruction. Detailed IPET reconstruction of particle No. 11 was presented here. **a,** Seven representative tilted views of an individual IgG antibody particle shown in the first column (SNR: 0.18), followed by the corresponding projections of 3D reconstruction from major iterations (second to fifth columns), with the final result from each tilting angle shown in the last column. **b,** The finalized 3D reconstruction image (SNR: 1.45) of a targeted individual antibody eventually obtained after the above process. **c,** 3D density map and pseudo structure. **d,** FSC curve of IPET reconstruction. **e,** Five snapshot images illustrated the ease of TMD simulation. Molecular Dynamic simulation was used to steer the three domains with a force calculated to match ultimately the three domain density maps in an aqueous environment. The scale bar was 50 Å.

Supplementary Figure 13 | Detailed procedures of the twelfth representative IPET reconstruction. Detailed IPET reconstruction of particle No. 12 was presented here. **a,** Seven representative tilted views of an individual IgG antibody particle shown in the first column (SNR: 0.24), followed by the corresponding projections of 3D reconstruction from major iterations (second to fifth columns), with the final result from each tilting angle shown in the last column. **b,** The finalized 3D reconstruction image (SNR: 1.84) of a targeted individual antibody eventually obtained after the above process. **c,** 3D density map and pseudo structure. **d,** FSC curve of IPET reconstruction. **e,** Five snapshot images illustrated the ease of TMD simulation. Molecular Dynamic simulation was used to steer the three domains with a force calculated to match ultimately the three domain density maps in an aqueous environment. The scale bar was 50 Å.

Supplementary Figure 14 | Detailed procedures of the thirteenth representative IPET reconstruction. Detailed IPET reconstruction of particle No. 120 was presented here. **a,** Seven representative tilted views of an individual IgG antibody particle shown in the first column (SNR: 0.26), followed by the corresponding projections of 3D reconstruction from major iterations (second to fifth columns), with the final result from each tilting angle shown in the last column. **b,** The finalized 3D reconstruction image (SNR: 1.50) of a targeted individual antibody eventually obtained after the above process. **c,** 3D density map and pseudo structure. **d,** FSC curve of IPET reconstruction. **e,** Five snapshot images illustrated the ease of TMD simulation. Molecular Dynamic simulation was used to steer the three

domains with a force calculated to match ultimately the three domain density maps in an aqueous environment. The scale bar was 50 Å.

Supplementary Figure 15 | Averaging the density maps of 120 3D density maps of IgG1 antibody. As a single-particle electron tomography method, all 120 IPET 3D density maps were aligned and averaged. The averaged map showed fuzzy domains as similar to those from the 2D class averages and single-particle reconstructions. All density maps were shifted and rotated with respect to the respective domain/linker positions described by vector map in a, and averaged.

Supplementary Video

Supplementary Video | Exploring 3D conformational flexibility of IgG1 antibody by IPET. A tilt series of EM tomography of IgG1 antibody by optimized negative-staining (OpNS). The tilt series was imaged by tilting angles of a range from -60° to -60° in steps of 1.5°. The particles marked by red boxes were submitted for individual-particle electron tomography (IPET) reconstruction. As an example, a target particle in a green box was zoomed-in for demonstrating the process of IPET 3D reconstruction. The movies were: the tilt series of raw images; projections of the intermediate 3D reconstruction with a soft-circular mask; projections of IPET final 3D density map with a particle-shaped soft-mask; isosurface of final 3D reconstruction; comparing to the crystal structure 1IGT after ridge-body docked the F_c domain into EM density map; TMD fitting, and final PDB structure. Using the IPET 3D reconstruction as a constraint, the crystal structure of the IgG1 antibody was flexibly docked into the IPET density maps, by targeted molecular dynamics (TMD) simulations, to achieve new conformations of the antibody. By repeating the above processes again and again on each target particle, we achieved the 120 3D reconstructions from 120 targeted antibody molecules, which contributed 120 conformations of this type of antibody. This morphing conformation reflected the antibody conformational flexibility and dynamics. By aligning the F_c domains of 120 conformations of antibodies together, it revealed that the F_{ab} domains varied in positions and orientations. The overall distribution of 120 conformations showed a hemi-ellipsoidal shape, reflecting the conformational flexibility and dynamics of the antibody.

Supplementary References

1. Harris, L.J., Larson, S.B., Hasel, K.W. & McPherson, A. Refined structure of an intact IgG2a monoclonal antibody. *Biochemistry* **36**, 1581-1597 (1997).

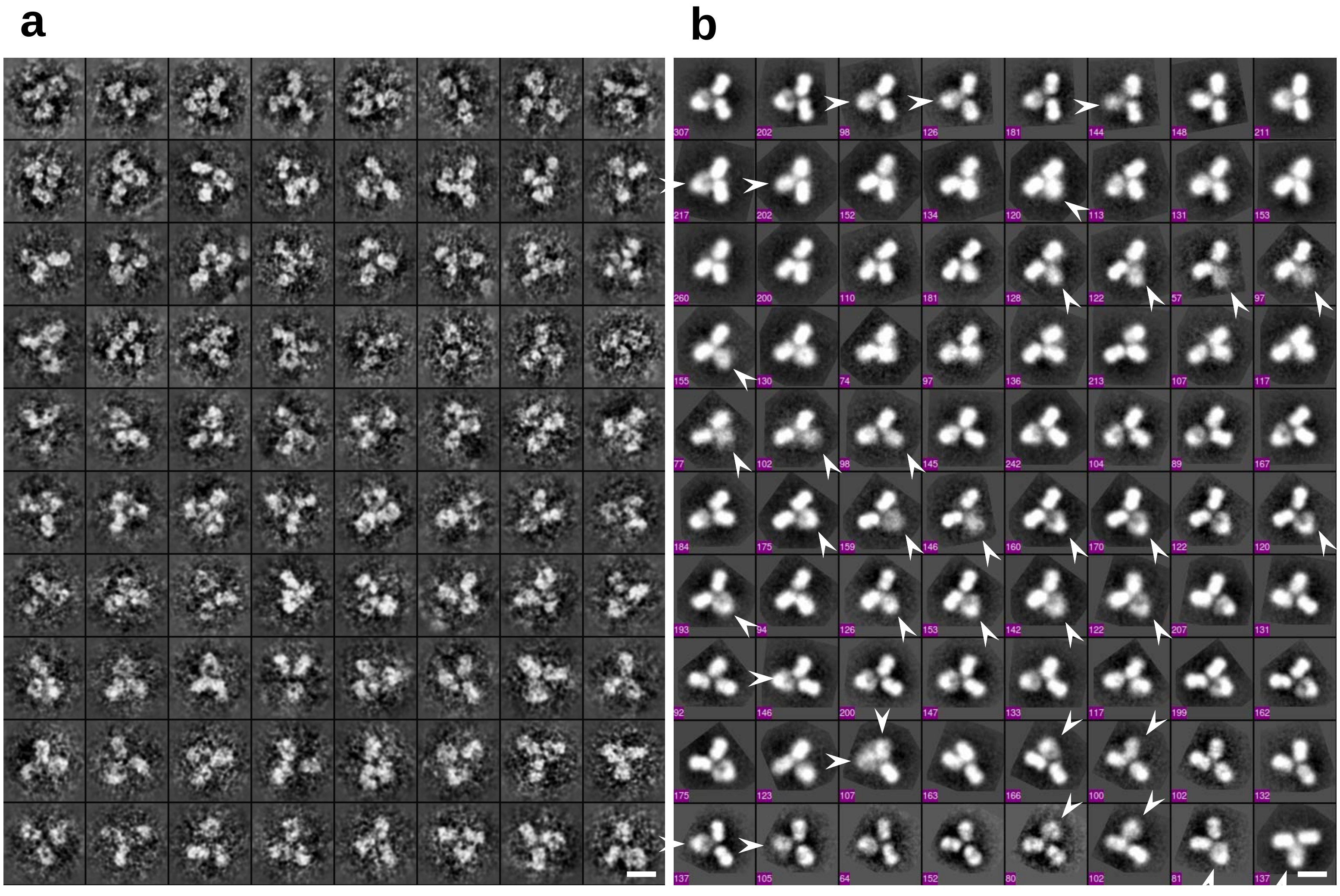

Supplementary Figure 1

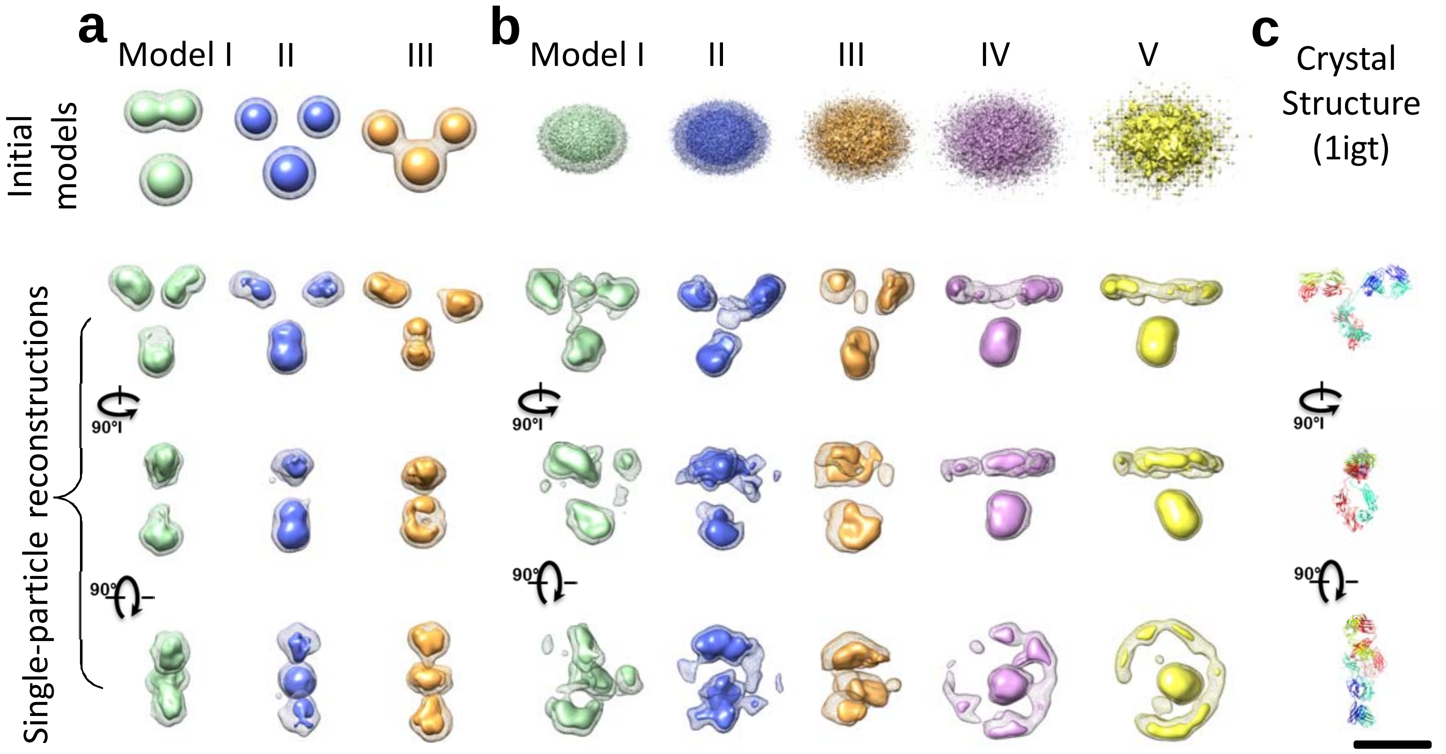

Supplementary Figure 2

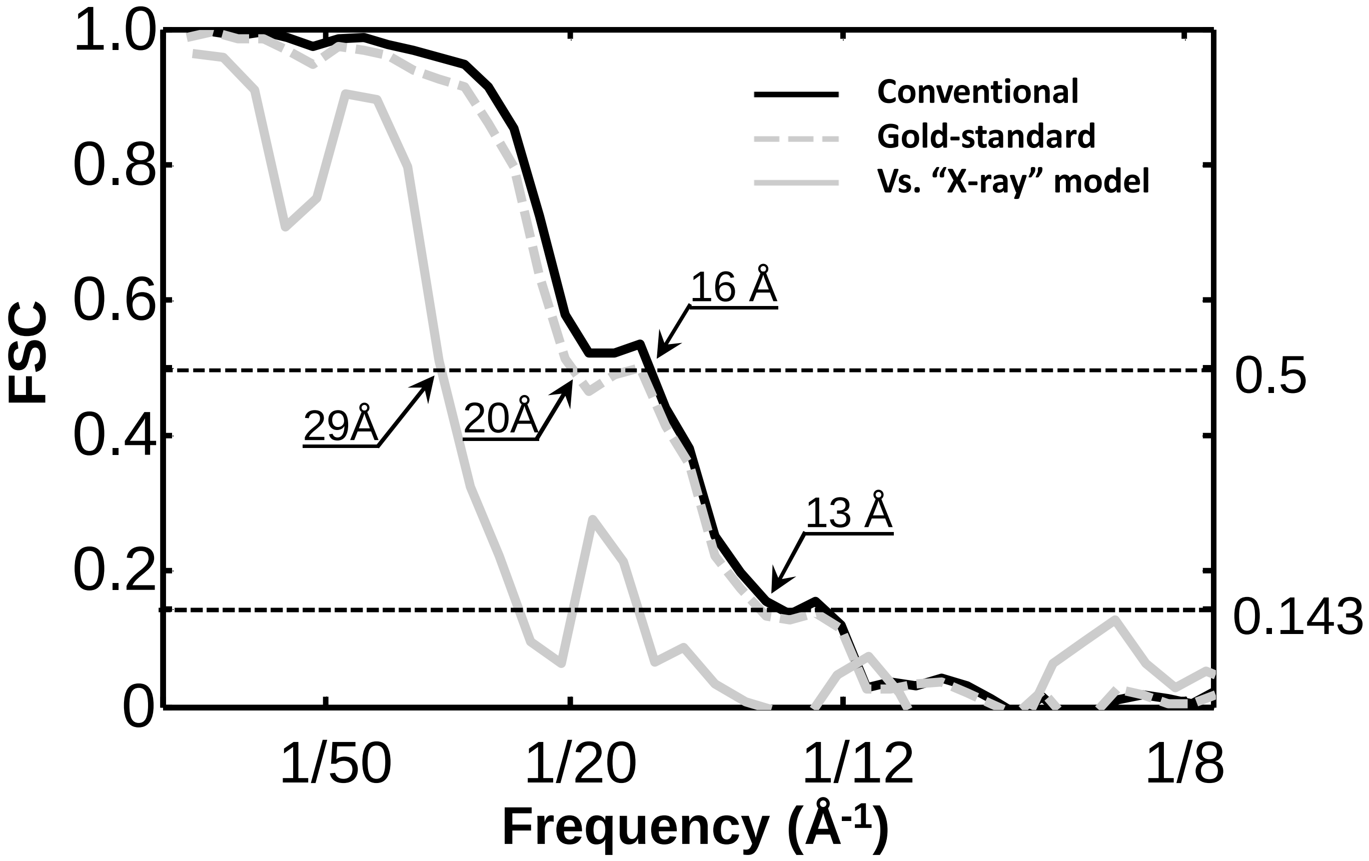

Supplementary Figure 3

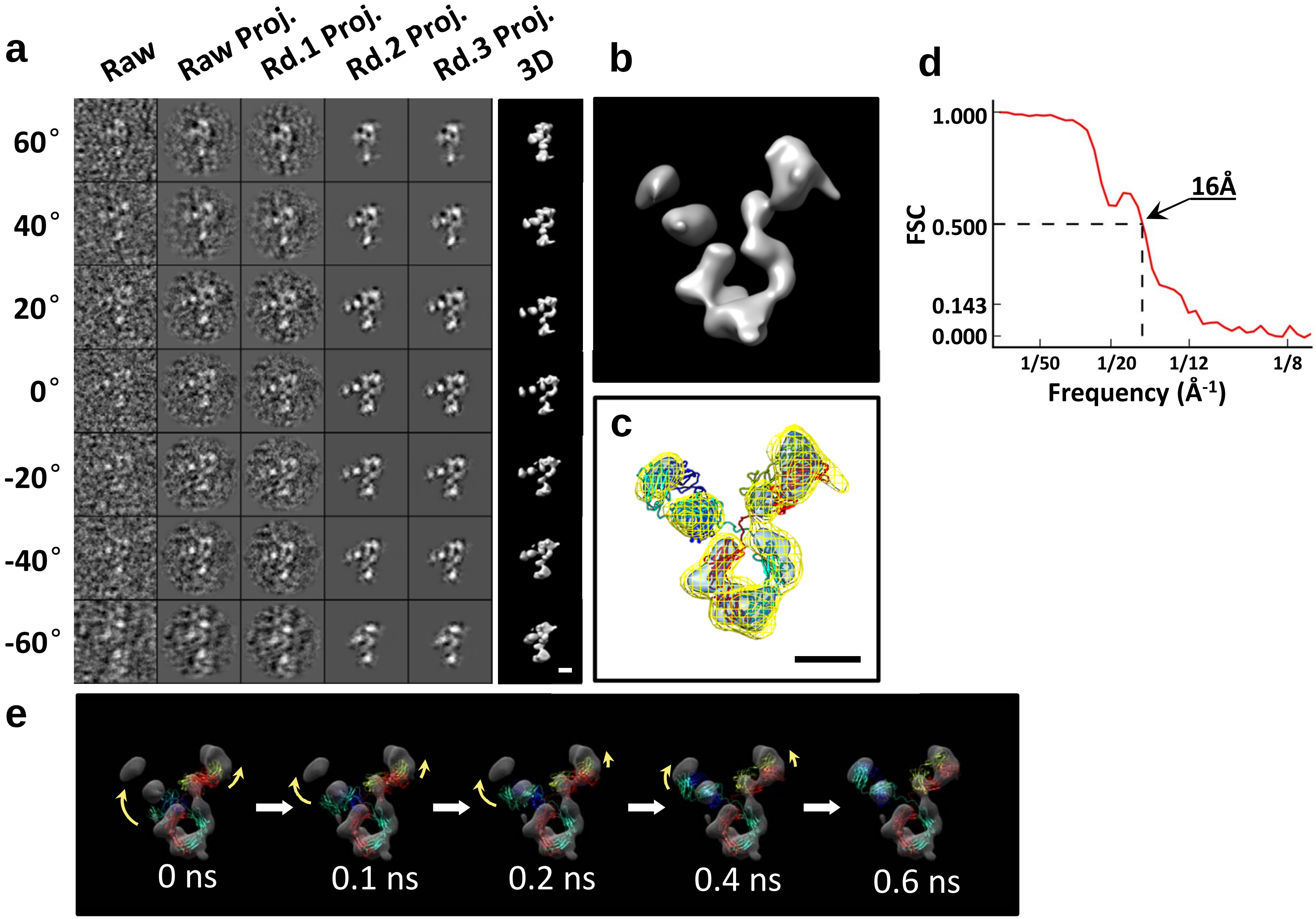

Supplementary Figure 4

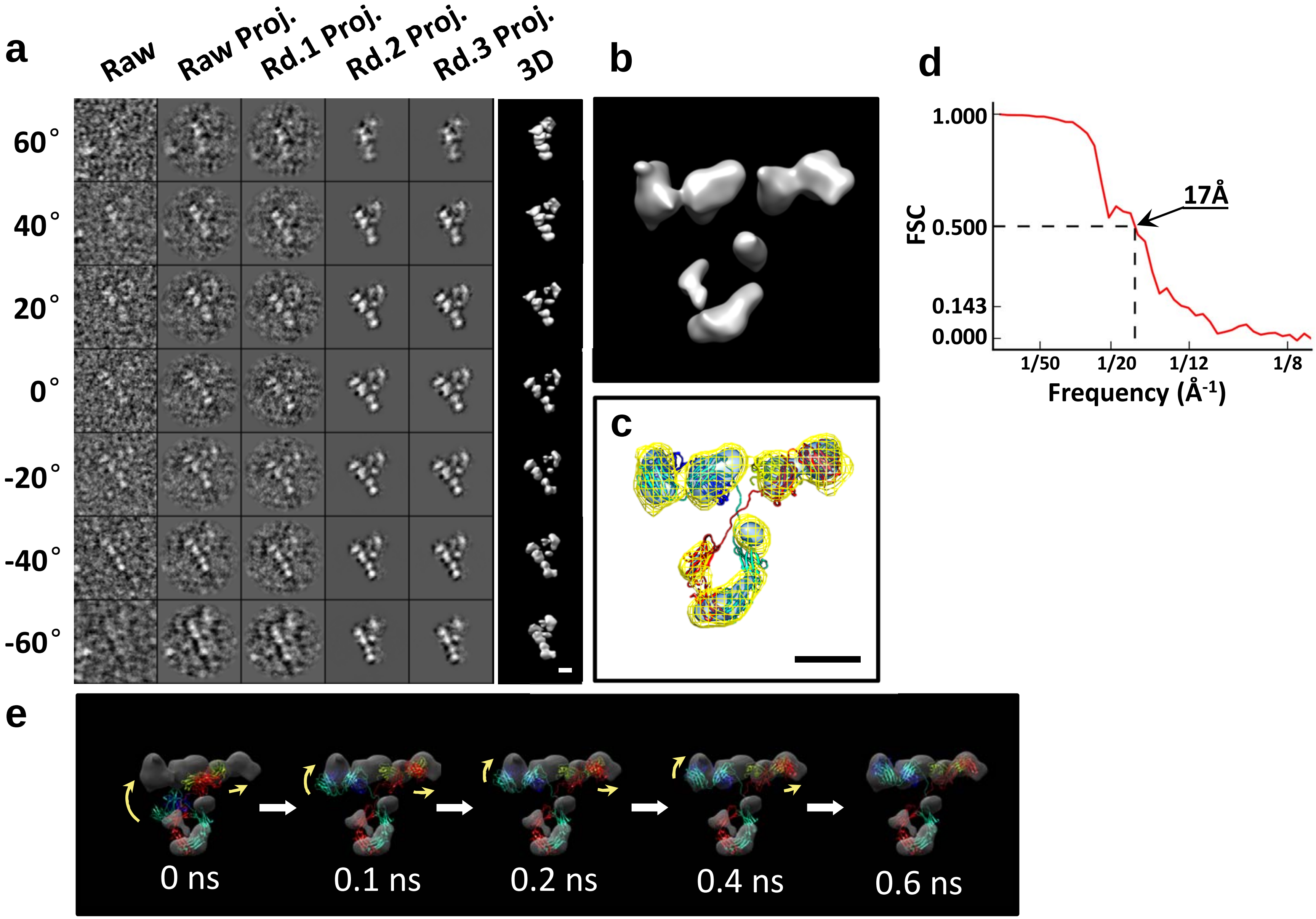

Supplementary Figure 5

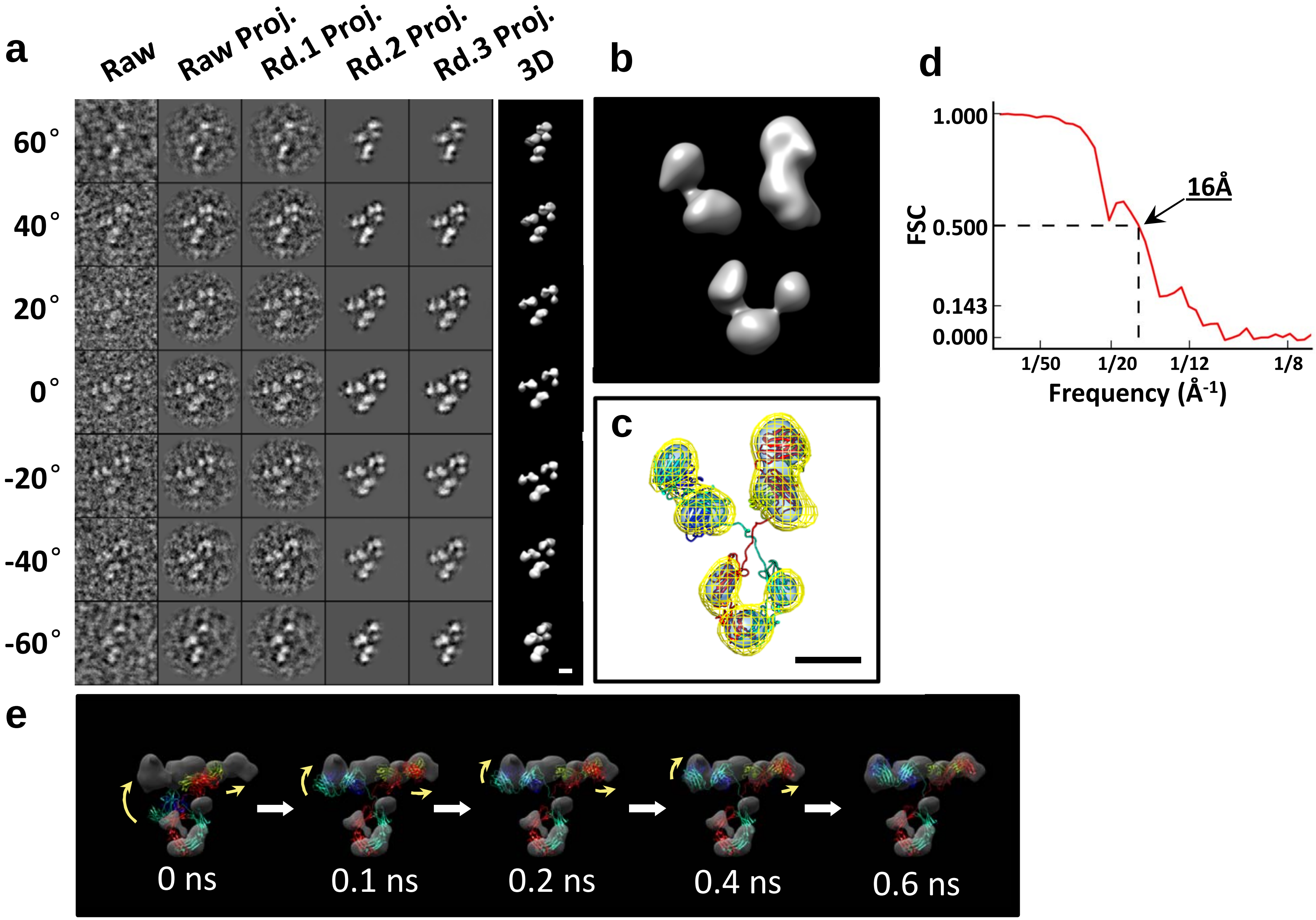

Supplementary Figure 6

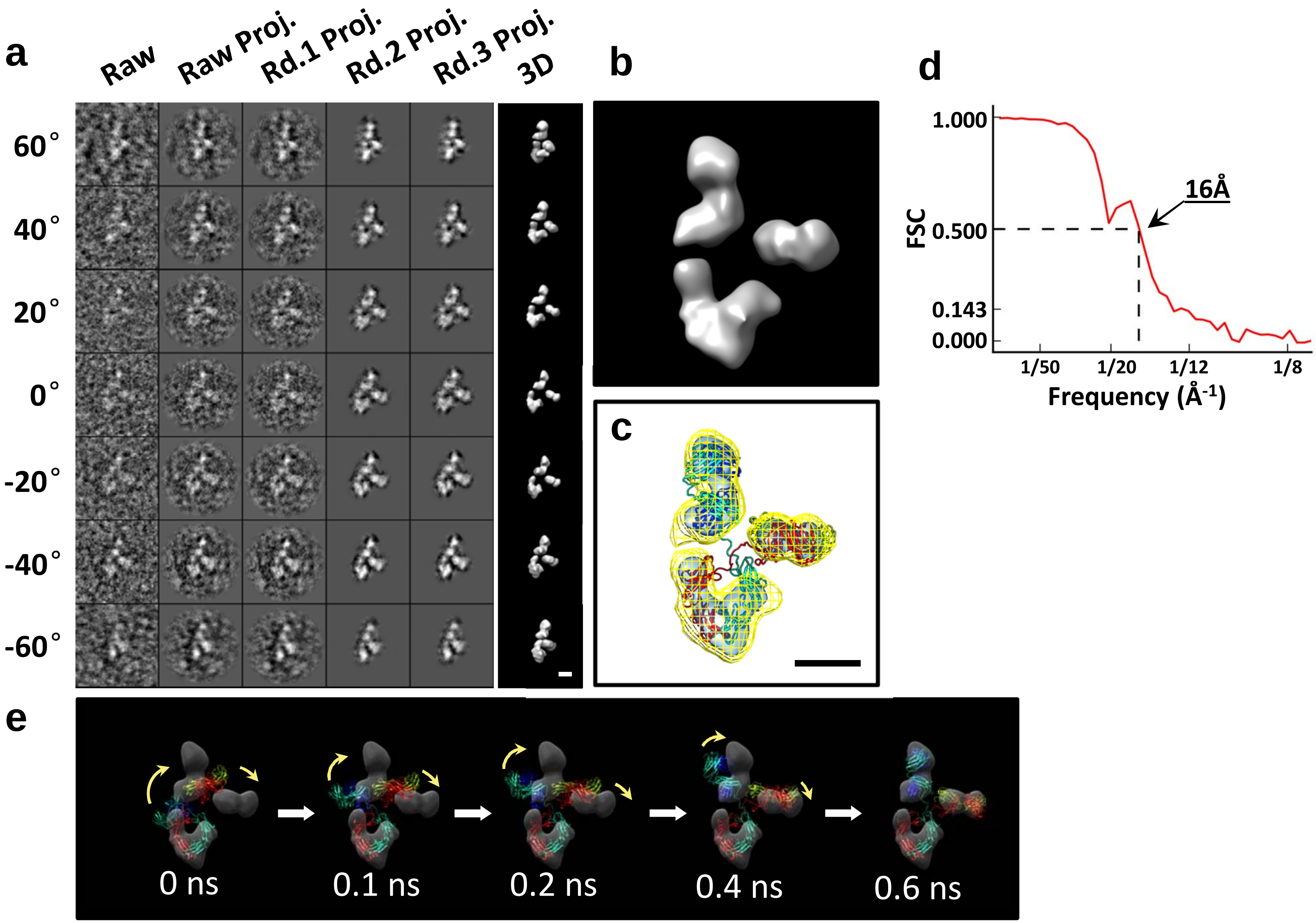

Supplementary Figure 7

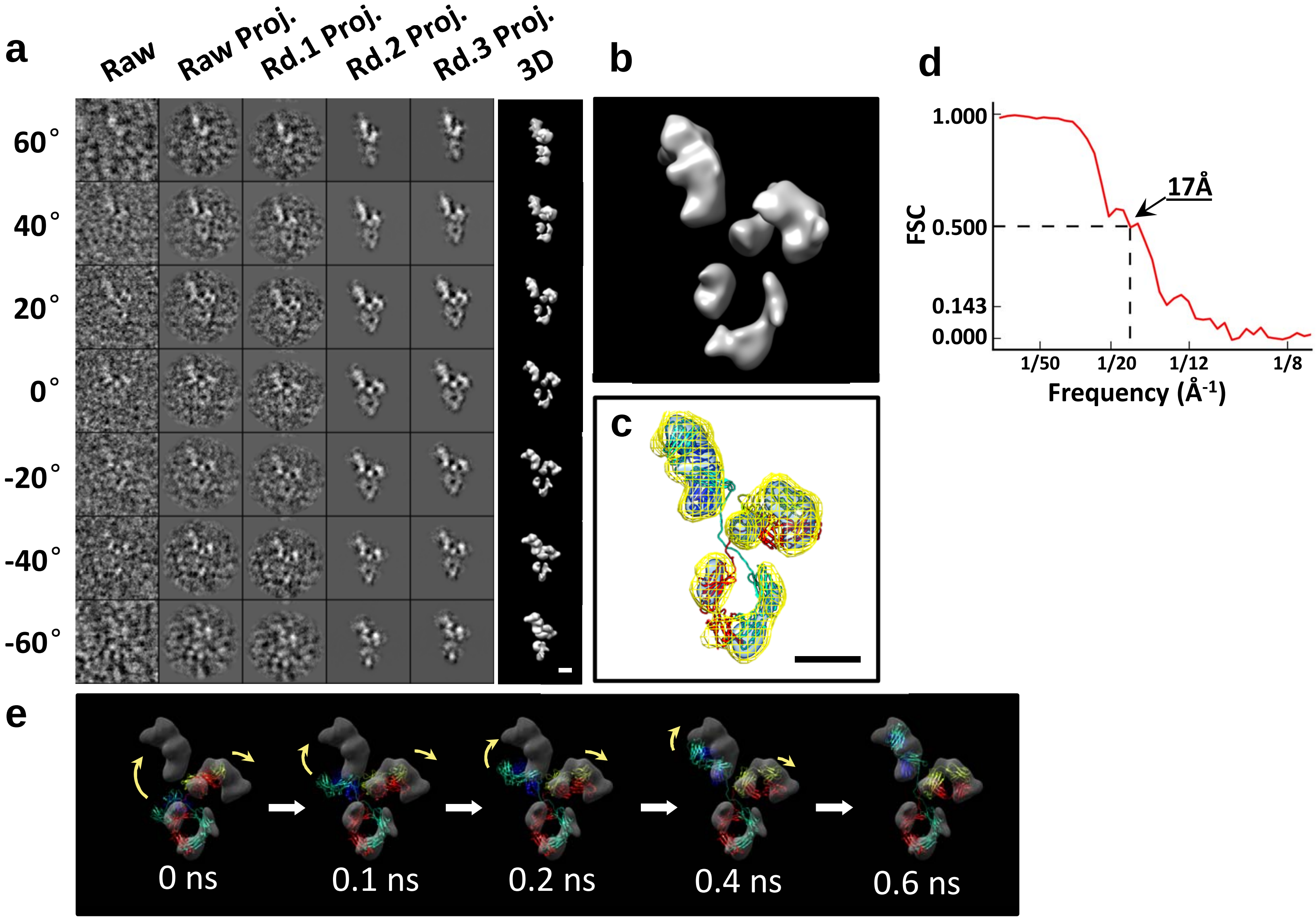

Supplementary Figure 8

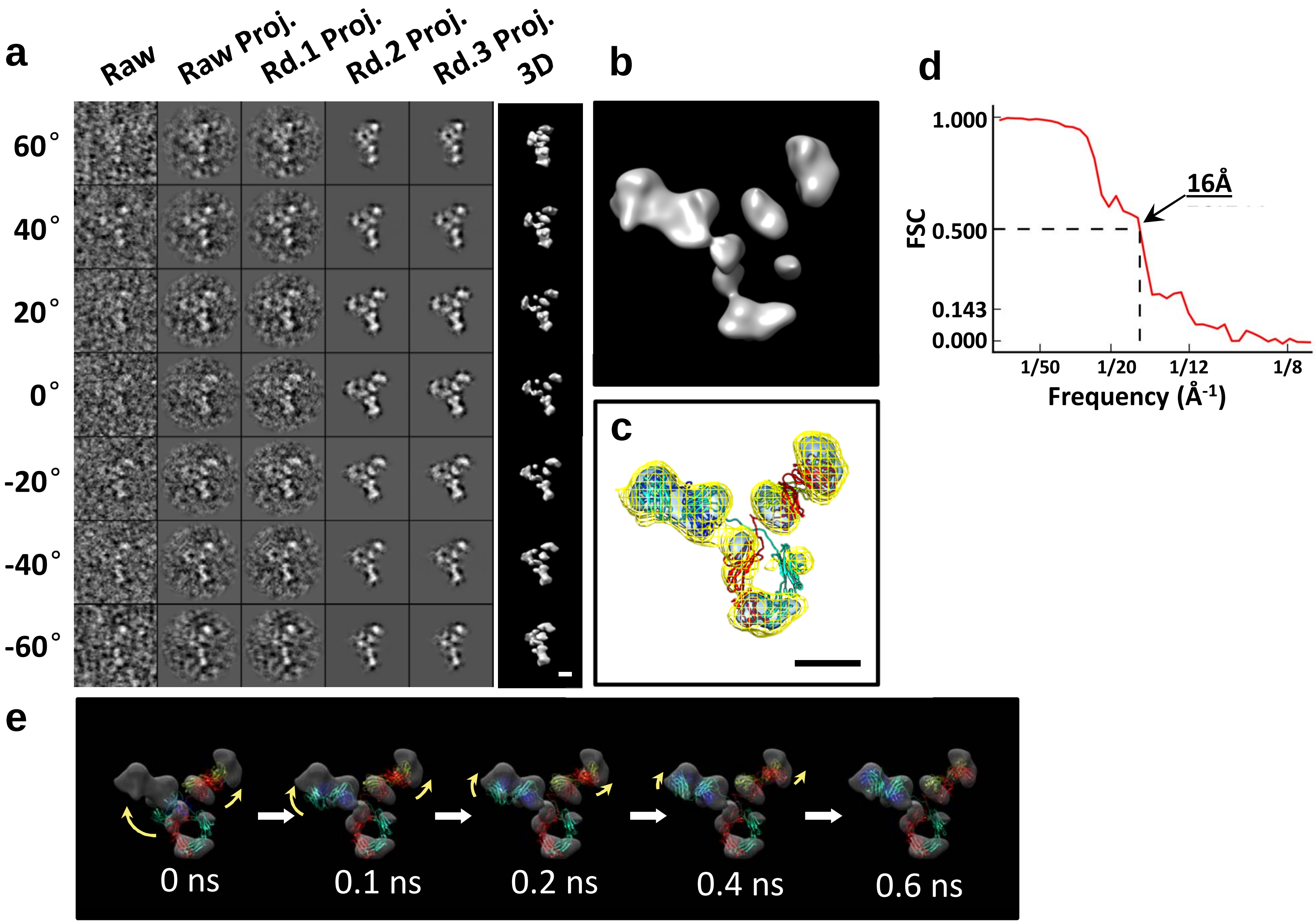

Supplementary Figure 9

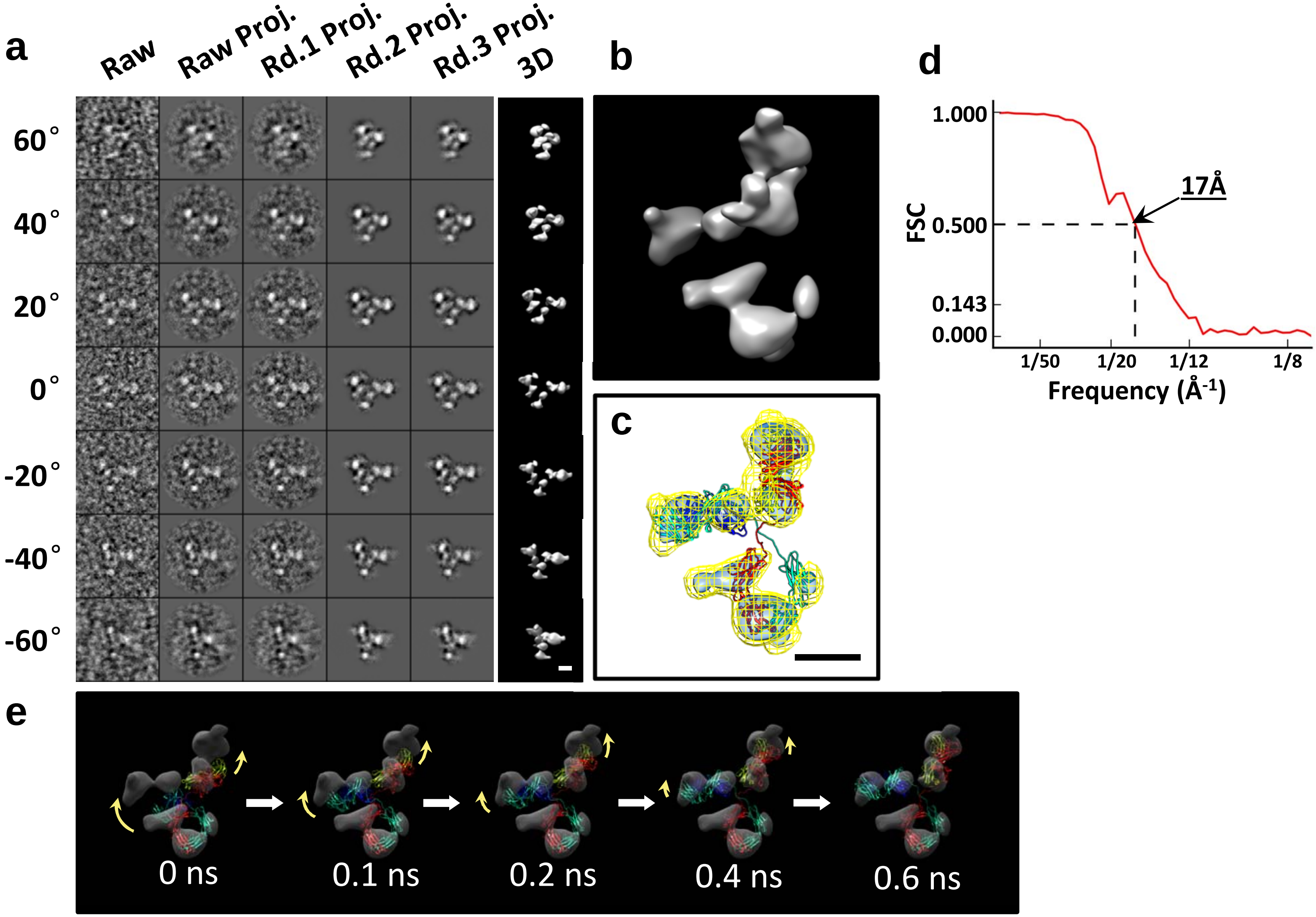

Supplementary Figure 10

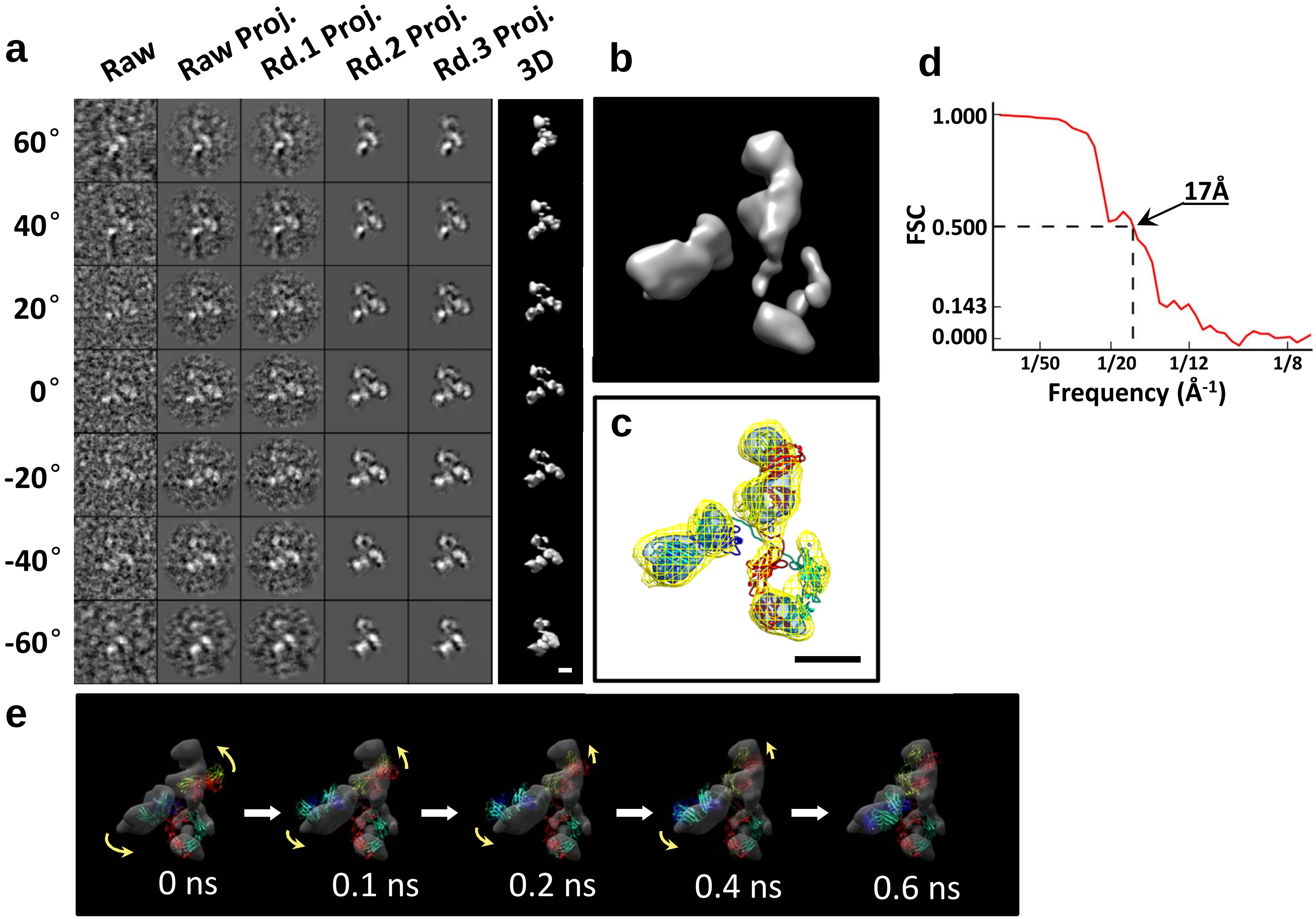

Supplementary Figure 11

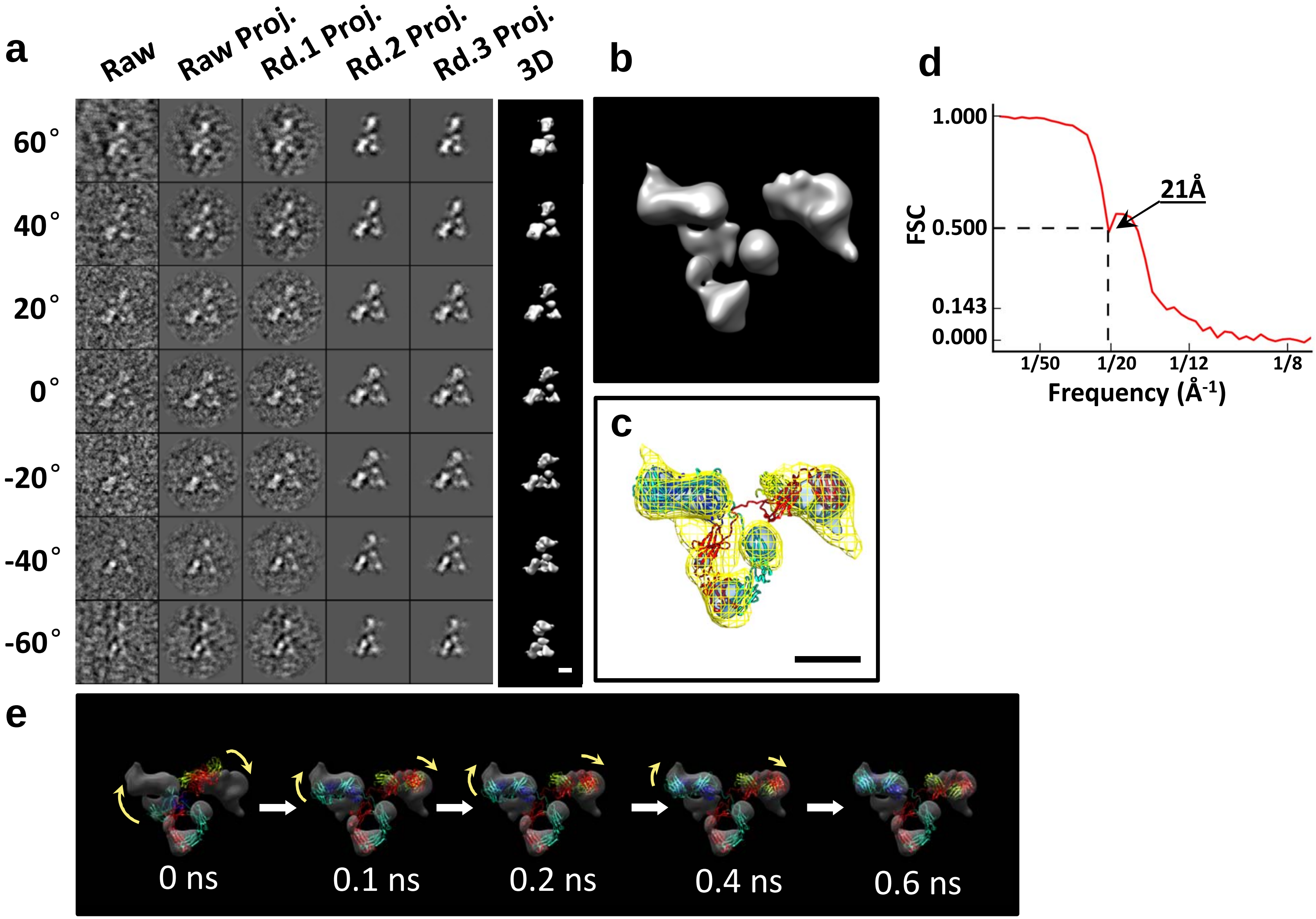

Supplementary Figure 12

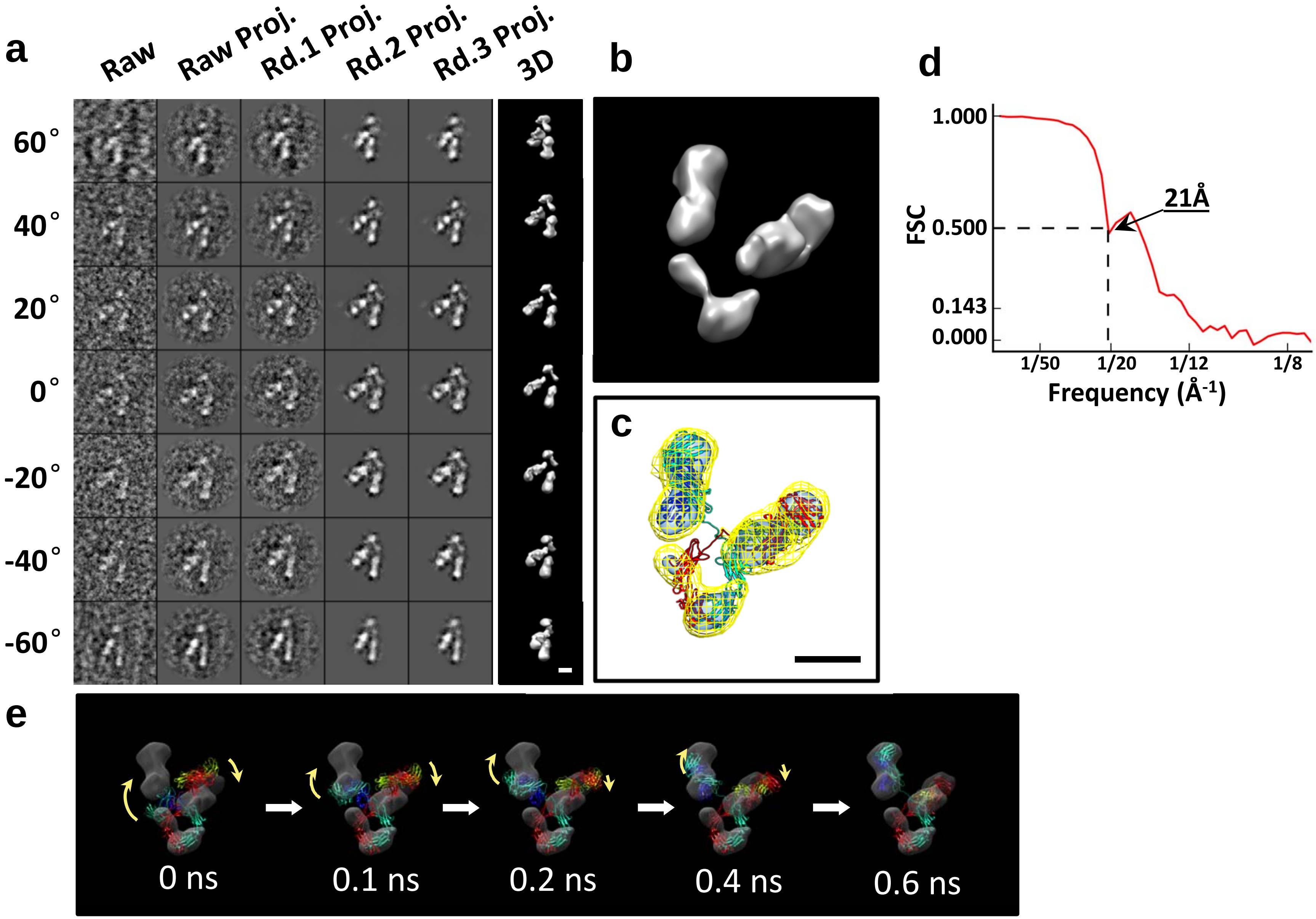

Supplementary Figure 13

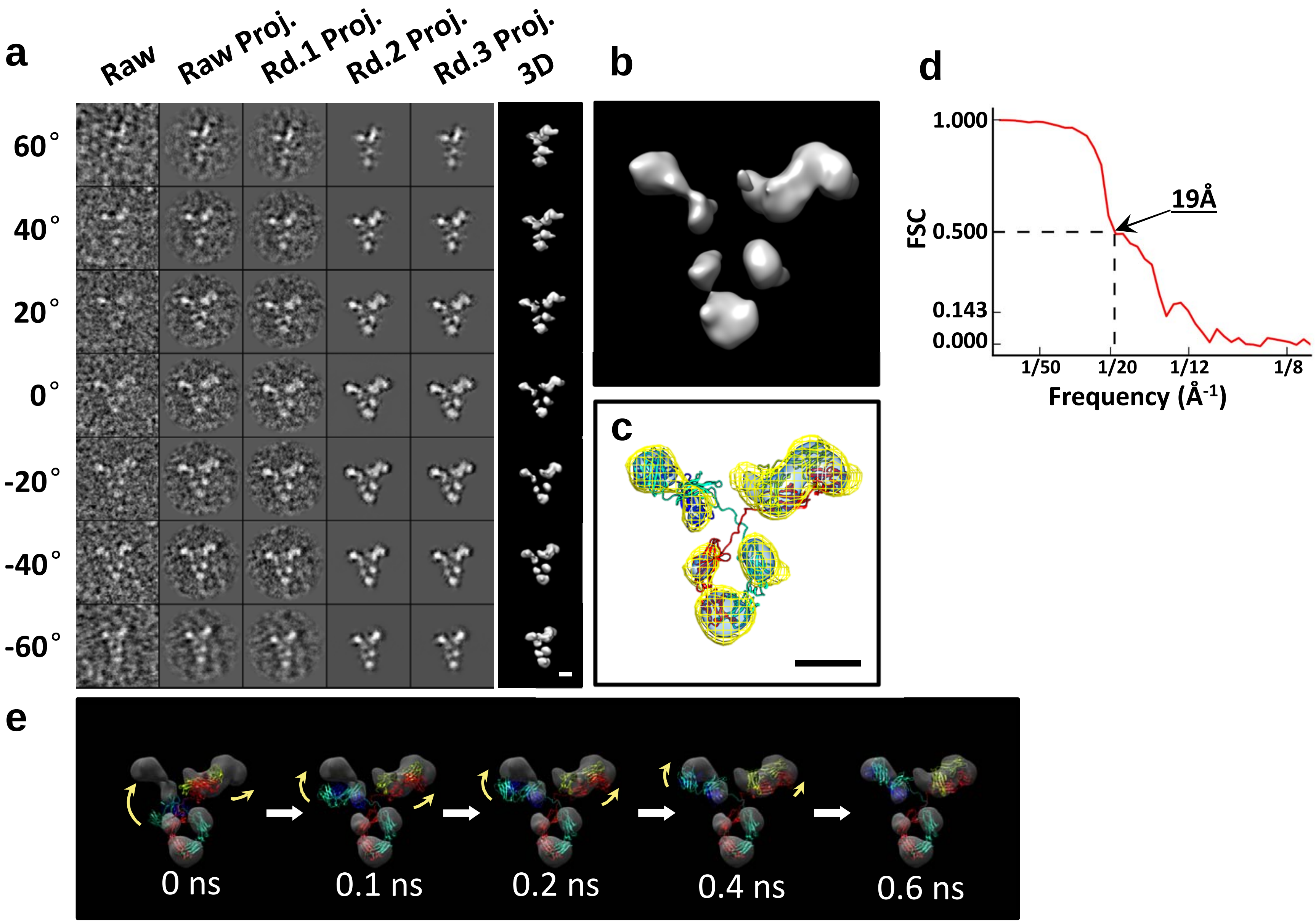

Supplementary Figure 14

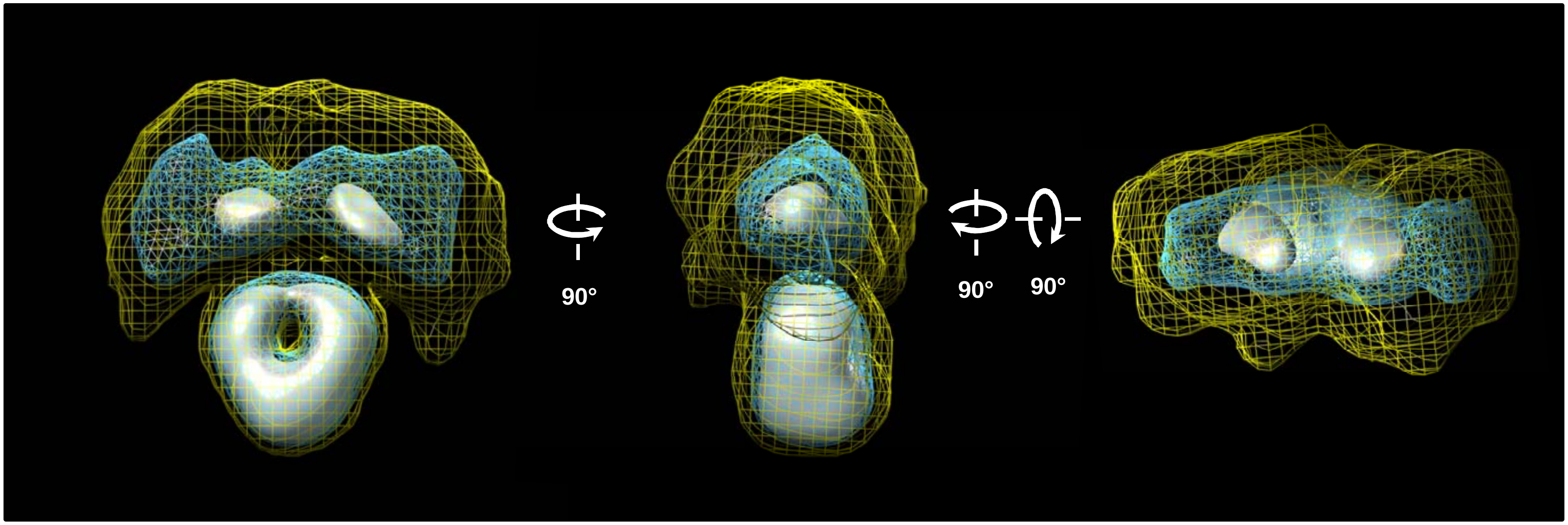

Supplementary Figure 15
